# Mirror-Image Organometallic Osmium Arene Iminopyridine Halido Complexes Exhibit Similar Potent Anticancer Activity

**DOI:** 10.1002/chem.201302183

**Published:** 2013-09-23

**Authors:** Ying Fu, Rina Soni, María J Romero, Ana M Pizarro, Luca Salassa, Guy J Clarkson, Jessica M Hearn, Abraha Habtemariam, Martin Wills, Peter J Sadler

**Affiliations:** [a] Department of Chemistry, University of WarwickGibbet Hill Road, Coventry, CV4 7AL (UK); [b] CIC biomaGUNEPaseo Miramón 182, 20009 Donostia-San Sebastián (Spain); [c] Warwick Systems Biology Centre, University of WarwickGibbet Hill Road, Coventry, CV4 7AL (UK)

**Keywords:** anticancer agents, arene ligands, chirality, organometallic, osmium

## Abstract

Four chiral Os^II^ arene anticancer complexes have been isolated by fractional crystallization. The two iodido complexes, (*S*_Os_,*S*_C_)-[Os(η^6^-*p*-cym)(ImpyMe)I]PF_6_ (complex **2**, (*S*)-ImpyMe: *N*-(2-pyridylmethylene)-(*S*)-1-phenylethylamine) and (*R*_Os_,*R*_C_)-[Os(η^6^-*p*-cym)(ImpyMe)I]PF_6_ (complex **4**, (*R*)-ImpyMe: *N*-(2-pyridylmethylene)-(*R*)-1-phenylethylamine), showed higher anticancer activity (lower IC_50_ values) towards A2780 human ovarian cancer cells than cisplatin and were more active than the two chlorido derivatives, (*S*_Os_,*S*_C_)*-*[Os(η^6^-*p*-cym)(ImpyMe)Cl]PF_6_, **1**, and (*R*_Os_,*R*_C_)-[Os(η^6^-*p*-cym)(ImpyMe)Cl]PF_6_, **3**. The two iodido complexes were evaluated in the National Cancer Institute 60-cell-line screen, by using the COMPARE algorithm. This showed that the two potent iodido complexes, **2** (NSC: D-758116/1) and **4** (NSC: D-758118/1), share surprisingly similar cancer cell selectivity patterns with the anti-microtubule drug, vinblastine sulfate. However, no direct effect on tubulin polymerization was found for **2** and **4**, an observation that appears to indicate a novel mechanism of action. In addition, complexes **2** and **4** demonstrated potential as transfer-hydrogenation catalysts for imine reduction.

## Introduction

The U.S. Food and Drug Administration (FDA) has defined strict rules for the development of new stereoisomeric drugs, especially following the tragedy of severe birth defects caused by the *S* isomer of thalidomide^1^ (originally developed as an antisedative drug, then found to be an inhibitor of angiogenesis for anticancer treatment^2^, ^3^). The fast-growing field of bioorganometallic chemistry has attracted much interest in the development of the next generation of anticancer agents following the success of the platinum-based drugs cisplatin, carboplatin, and oxaliplatin.^4^–^8^ Examples include ruthenium and osmium organometallic complexes that show promising anticancer activity.^9^–^18^ Notably, a number of these organometallic complexes contain chiral metal centers.^13^–^18^ Although there are no preclinical or clinical reports of different activity of specific stereoisomers of an organometallic complex, it is of interest to investigate the possible role of stereochemistry in the biological activity for this class of compounds. In particular, the design of metal-based anticancer agents now also includes those interacting with protein targets, a fact that requires careful control of the chirality on the metal center.^19^, ^20^ For example, the chirality of a ruthenium center has been shown to affect the inhibition of glycogen synthase kinase 3β.^21^ However, such studies are scarce, probably owing to the difficulty of isolating enantiomers or diastereomers of organometallic complexes.^22^, ^23^

The first examples of organometallic complexes isolated with defined chiral metal centers appear to be those reported in 1969 by Brunner:^24^ [M(η^5^-C_5_H_5_)(CO)(NO)(Ph_3_P)] (M=Cr, Mo, W) and [Mn(η^5^-C_5_H_5_)(CO)(NO)(Ph_3_P)]PF_6_. These complexes are all configurationally stable in the solid state. The configurational stability of the metal center in solution depends on the monodentate ligand; for example, (*R*_Mn_,*R*_C_)- and (*S*_Mn_,*R*_C_)-[Mn(η^5^-C_5_H_5_)(CO)(NO)(Ph_3_P)]PF_6_ are configurationally stable, whereas (*R*_Mn_,*R*_C_)- and (*S*_Mn_,*R*_C_)- [Mn(η^5^-C_5_H_5_)(COR)(NO)(Ph_3_P)]PF_6_ (R=acyl) can epimerize.^25^ Other factors involved in the configurational lability at the metal center in solution, such as temperature, solvent, or structural features, have been analyzed for diastereomeric Ru^II^ organometallic arene complexes,^26^–^28^ but fewer studies have been carried out on epimerization of Os^II^ arene diastereomers.^29^ Examples of enantiopure half-sandwich anticancer complexes are scarce in the literature.^30^, ^31^ In particular, the biological properties of pure epimers of chiral-at-osmium arene complexes have not been reported to date.

Os^II^,^32^–^34^ Os^III^,^35^ Os^IV[36]^ and Os^VI[37]^ complexes have been reported to show promising anticancer activity in recent years. Half-sandwich Os^II^ organometallic arene anticancer complexes containing a monodentate ligand and an unsymmetrical chelating ligand are chiral. Previously, we reported that the synthesis of the anticancer Os^II^ arene iminopyridine (Impy) complex, [Os(η^6^-*p*-cym)(Impy-OH)I]PF_6_ (*p*-cym= *para*-cymene, Impy-OH=4-[(2-pyridinylmethylene)amino]-phenol), gives a mixture of enantiomers in approximately 1:1 ratio.^14^ The enantiomeric resolution of such organometallic arene complexes would also be interesting in terms of elucidating mechanisms of action. Nevertheless, the purification of chiral osmium isomers is not easily achieved as chiral columns often produce low yields, and selective chiral synthesis on a metal center is still not readily achievable. Introduction of a chiral carbon atom into the chelated iminopyridine ligand of Os^II^ arene complexes has allowed a facile separation by fractional crystallization of the resulting diastereomeric complexes, which have different physical properties.

Organometallic arene iminopyridine complexes containing ruthenium^38^, ^39^ and iridium^40^ can also act as transfer-hydrogenation catalysts. Catalytic transfer hydrogenation is a useful method for the preparation of amines of biological and chemical interest.^41^ The herein-reported Os^II^ arene diastereomers could be also attractive as asymmetric catalysts. There are very few reports on the catalytic potential of Os^II^ arene complexes as transfer-hydrogenation catalysts for imine reduction.^42^, ^43^ Thus, the four Os^II^ arene iminopyridine complexes were also investigated as transfer-hydrogenation catalysts^42^, ^43^ using a model imine substrate.

## Results

The pure chiral iminopyridine ligand ((*S*)*-* or (*R*)-ImpyMe: *N*-(2-pyridylmethylene)-(*S*)-1-phenylethylamine or *N*-(2-pyridylmethylene)-(*R*)-1-phenylethylamine) was reacted with the Os^II^ dimer—[{Os(η^6^-*p*-cym)Cl_2_}_2_] or [{Os(η^6^-*p*-cym)I_2_}_2_]—to form both diastereomers; the diastereomer that crystallized first was collected from each reaction and the second diastereomer was left in the mother liquor. Only isolated single crystals were used for the physical and biological studies of all the four osmium complexes reported in this work. Although this approach to the study of chirality has been widely used on Ru^II^ and Os^II^ arene catalysts with “piano-stool” geometry, there appears to be no report of any application in metallo-drug research. This approach could pave the way for further investigations on the effect of chirality of osmium metal centers on their pharmacological behavior, including metabolism, toxicity, tissue distribution, and excretion kinetics.^44^

**Scheme 1:**
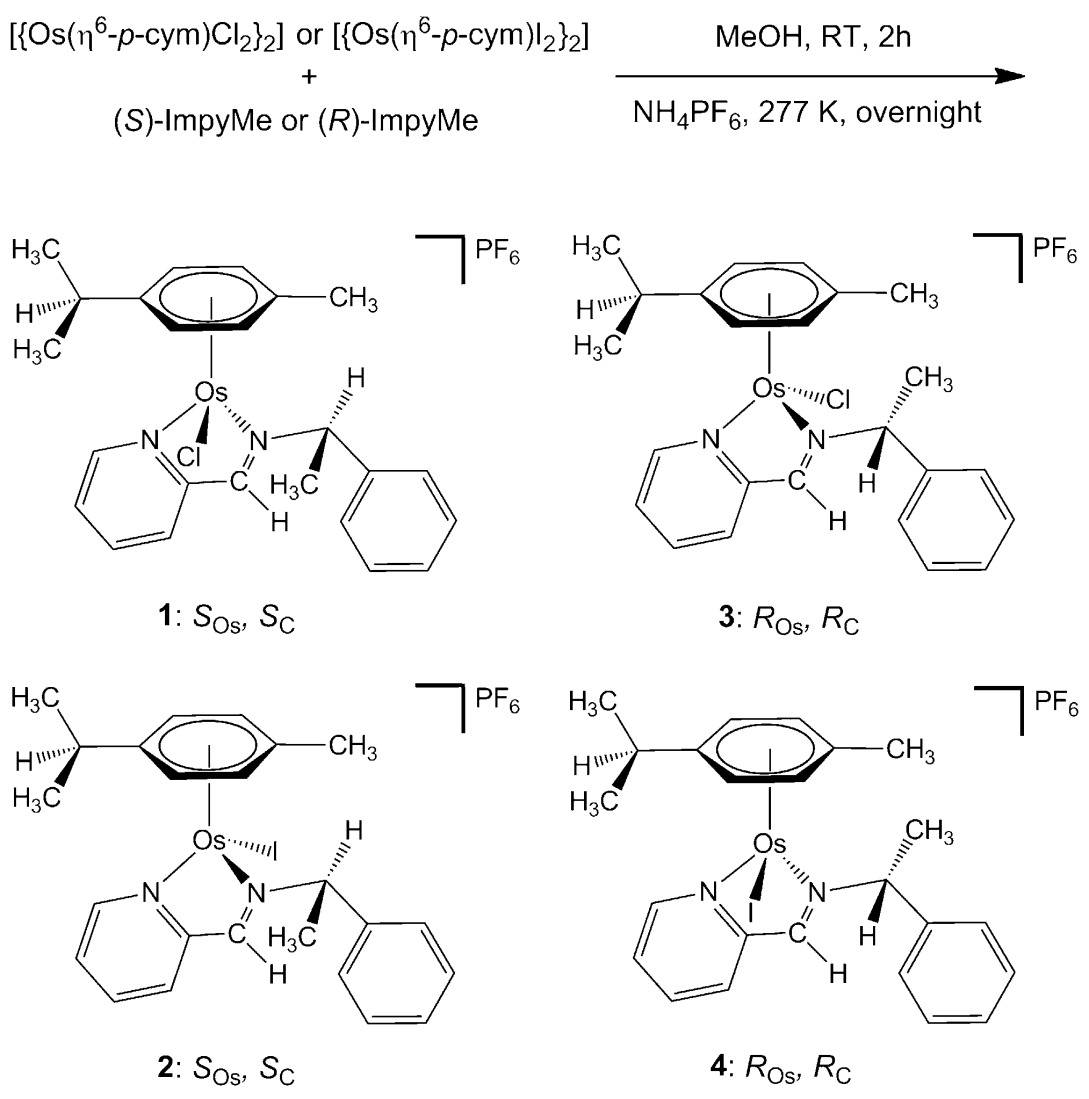
Synthetic route for the chiral Os^II^ complexes used in this work.

**Synthesis and characterization**: Two asymmetric imine ligands (*S*)- and (*R*)-ImpyMe containing a chiral carbon atom were synthesized by condensation of 2-pyridine carboxaldehyde with (*S*)-(−)-α-methylbenzylamine or (*R*)-(+)-α-methylbenzylamine and were purified by distillation by following a reported method.^45^ Four Os^II^ arene iminopyridine complexes of general formula [Os(η^6^-*p*-cym)(ImpyMe)X]PF_6_ (X=Cl, I) were synthesized by reaction of the corresponding dimer [{Os(η^6^-*p*-cym)X_2_}_2_] (X=Cl, I) and the chiral ligands (*S*)- or (*R*)-ImpyMe as described in the Experimental Section. As the chiral configuration of each enantiopure ligand is retained in solution, the Os^II^ arene complexes were obtained as a mixture of two diastereomers differing only in the metal configuration (*R*_Os_ or *S*_Os_). The isolation of diastereomerically pure complexes was accomplished by a crystallization method (Scheme 1). Thus, chiral-at-Os^II^ iminopyridine chlorido and iodido complexes **1**, **2**, **3**, and **4** were isolated as single crystals grown overnight at 277 K from a concentrated methanol solution. Unfortunately, the second diastereomer expected from each reaction could not be isolated as a pure compound although its formation was confirmed in the case of compound **3**. All four pure chiral Os^II^ iminopyridine complexes were characterized by CHN analysis, X-ray diffraction, ESI^+^ MS, ^1^H NMR and circular dichroism (CD) spectroscopy and their stability in aqueous solution was confirmed before screening for the anticancer activity.

**Figure 1:**
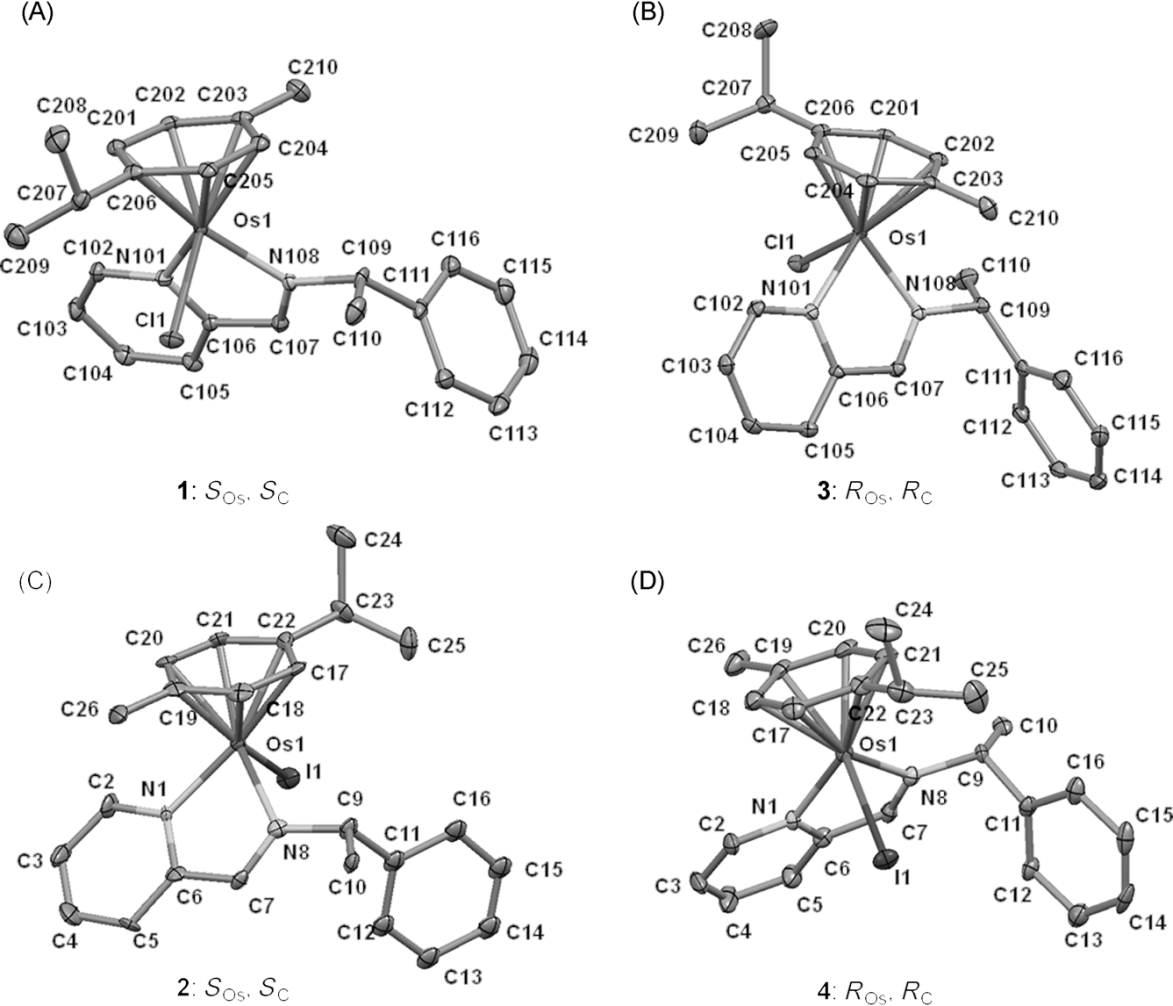
X-ray crystal structures of 1 (A), 2 (C), 3 (B), and 4 (D). Thermal ellipsoids are shown at 50 % probability. The hydrogen atoms and counterion have been omitted for clarity.

**X-ray diffraction**: The molecular structures of the Os^II^ arene iminopyridine complexes **1**, **2**, **3**, and **4** were established by X-ray crystallography. Only one pure diastereomer was observed in the unit cell of each compound and therefore the chirality on the osmium center could be determined unambiguously. The structures, along with their atom numbering schemes, are shown in Figure 1 A–D. Selected bond lengths and angles are listed in Tables 1 and 2. X-ray crystallographic data are reported in Table 3; the data show all the complexes crystallized in the same monoclinic space group: *P*2_1_. The complexes adopt the expected pseudo-octahedral “piano-stool” geometry with the osmium center bound to the arene ligand through η^6^ bonding (Os–arene centroid 1.682–1.695 Å). Additionally, Os^II^ is bound to a chloride (2.3892(7)–2.3913(8) Å) or iodide (2.7068(6)–2.7078(4) Å) and two nitrogen atoms (2.074(5)–2.124(9) Å) of the chelating ligand through σ bonds, these ligands constitute the three-legged structure of the “piano stool”. All the bond lengths and angles are in agreement with analogous osmium complexes previously reported.^46^

**Table 1:** Selected bond lengths [Å] and angles [°] for complexes 1 and 3.

	1(*S*_Os_,*S*_C_)[a]	3(*R*_Os_,*R*_C_)[b]
Os(1)–N(101) [Å]	2.082(3)	2.086(2)
Os(1)–N(108) [Å]	2.084(2)	2.086(2)
Os(1)–arene centroid [Å]	1.685	1.682
Os(1)–Cl(1) [Å]	2.3913(8)	2.3892(7)
N(101)-Os(1)-N(108) [°]	76.61(10)	76.43(9)

[a] Non-classical hydrogen-bond interactions for complex **1**: C102–H10A⋅⋅⋅F12, 2.48 Å [*x*, *y*, 1+*z*]; C105–H10D⋅⋅⋅F14, 2.44 Å [1+*x*, *y*, 1+*z*]; C109–H10F⋅⋅⋅F13, 2.37 Å; C202–H20B⋅⋅⋅F15, 2.40 Å; C205–H20D⋅⋅⋅F14, 2.43 Å [*x*, *y*, 1+*z*].

[b] Non-classical hydrogen-bond interactions for complex **3**: C102–H10A⋅⋅⋅F12, 2.48 Å [*x*, *y*, −1+*z*]; C105–H10D⋅⋅⋅F14, 2.44 Å [−1+*x*, *y*, −1+*z*], C109–H10F⋅⋅⋅F13, 2.38 Å; C202–H20B⋅⋅⋅F15, 2.39 Å; C205−H20D⋅⋅⋅F14, 2.43 Å [*x*, *y*, −1+*z*].

**Table 2:** Selected bond lengths [Å] and angles [°] for complexes 2 and 4.

	2(*S*_Os_,*S*_c_)[a]	4(*R*_Os_,*R*_c_)[b]
Os(1)–N(1) [Å]	2.084(6)	2.090(4)
Os(1)–N(8) [Å]	2.090(7)	2.074(5)
Os(1)–arene centroid [Å]	1.689	1.695
Os(1)–I(1) [Å]	2.7068(6)	2.7078(4)
N(1)-Os(1)-N(8) [°]	75.9(3)	76.17(17)

[a] Non-classical hydrogen-bond interactions for complex **2**: C4–H4A⋅⋅⋅F12, 2.37 Å [−1+*x*, *y*, *z*]; C7–H7A⋅⋅⋅F13A, 2.30 Å [−1+*x*, *y*, −1+*z*].

[b] Non-classical hydrogen-bond interactions for complex **4**: C4–H4A⋅⋅⋅F12, 2.37 Å [1+*x*, *y*, *z*]; C7–H7A⋅⋅⋅F13A, 2.31 Å [1+*x*, *y*, 1+*z*].

**Table 3:** X-ray crystallographic data and structure refinement for complexes 1, 3 and 2, 4.

	1(*S*_Os_,*S*_c_)	3(*R*_Os_,*R*_c_)	2(*S*_Os_,*S*_c_)	4(*R*_Os_,*R*_c_)
formula	C_24_H_28_ClF_6_N_2_OsP	C_24_H_28_ClF_6_N_2_OsP	C_24_H_28_F_6_IN_2_OsP	C_24_H_28_F_6_IN_2_OsP
*M*_r_	715.1	715.1	806.55	806.55
crystal system	monoclinic	monoclinic	monoclinic	monoclinic
crystal size [mm]	0.25×0.18×0.16	0.24×0.20×0.20	0.18×0.18×0.06	0.35×0.35×0.12
space group	*P*2_1_	*P*2_1_	*P*2_1_	*P*2_1_
crystal	brown block	orange block	brown block	brown block
*a* [Å]	10.19773(12)	10.19210(18)	9.1232(2)	9.1232(2)
*b* [Å]	11.79001(13)	11.78829(18)	15.0439(4)	15.0439(4)
*c* [Å]	10.99917(13)	10.99531(18)	9.6304(2)	9.6304(2)
*α* [°]	90	90	90	90
*β* [°]	103.0453(12)	103.0937(17)	97.591(3)	97.591(3)
*γ* [°]	90	90	90	90
*T* [K]	100(2)	100(2)	100(2)	100(2)
*Z*	2	2	2	2
*μ* [mm^−1^]	5.174	5.181	6.164	6.164
reflections collected	27 544	11 484	13 060	12 315
independent reflections [*R*_int_]	8081 [0.0360]	6327 [0.0204]	6754 [0.0526]	6765 [0.0294]
*R*_1_, *wR*_2_ [*F*>4*σ*(*F*)][a,b]	0.0241, 0.0517	0.0184, 0.0387	0.0427, 0.0951	0.0280, 0.0691
*R*_1_, *wR*_2_ (all data)[a,b]	0.0260. 0.0526	0.0192, 0.0391	0.0522, 0.0989	0.0288, 0.0698
GOF[c]	1.017	1.022	1.02	1.03
Δ*ρ* max/min [e Å^−3^]	1.217 and −0.796	1.046 and −0.718	2.220 and −1.676	1.904 and −1.058

[a] *R*_1_=Σ||*F*_o_|−|*F*_c_||/Σ|*F*_o_|.

[b]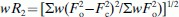.

[c]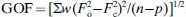.

Cahn–Ingold–Prelog priority rules (CIP system) cannot be applied directly to pseudo-four-coordinate organometallic chiral-at-metal arene complexes. Therefore, to assign the chirality in the *R* and *S* convention for these Os^II^ arene complexes, the modified CIP rules for organometallic arene complexes suggested by Tirouflet et al.^47^ and Stanley and Baird^48^ were used; the *p*-cymene arene (η^6^-C_6_) was considered as a pseudo-atom with atomic weight 72. We defined the priority sequence of ligands attached to the Os^II^ center as follows:^47^, ^48^ η^6^-C_6_>Cl>N (imine)>N (pyridine) or I>η^6^-C_6_>N (imine)>N (pyridine). According to the sequence rule of the *R*/*S* system, the configurations of the Os^II^ centers in these four chiral osmium arene iminopyridine complexes are: **1**=*S*_Os_, **2**=*S*_Os_, **3**=*R*_Os_, and **4**=*R*_Os_. Therefore, the retention of chirality at osmium between each chlorido complex and its iodido analogue is just a consequence of the change in the priority sequence, as an inversion of configuration at the metal center is observed in the crystal structures (Figure 1 A–D).

The four complexes can be divided into two enantiomeric pairs according to the different monodentate ligand coordinated to osmium (chloride or iodide): (*S*_Os_,*S*_C_)*-*[Os(η^6^-*p*-cym)(ImpyMe)Cl]PF_6_ (**1**) and (*R*_Os_,*R*_C_)*-*[Os(η^6^-*p*-cym)(ImpyMe)Cl]PF_6_ (**3**), (*S*_Os_,*S*_C_)*-*[Os(η^6^-*p*-cym)(ImpyMe)I]PF_6_ (**2**) and (*R*_Os_,*R*_C_)*-*[Os(η^6^-*p*-cym)(ImpyMe)I]PF_6_ (**4**). The comparison between the crystal structures of both pairs of complexes showed no significant differences in their bond lengths and angles around the osmium center.

**^1^H NMR spectroscopy**: The ^1^H NMR spectra of **1**, **2**, **3**, and **4** in [D_6_]acetone were recorded at 298 K. Identical ^1^H NMR data (see Experimental Section) were obtained for both of the chlorido complexes, **1** and **3**, as well as for both of the iodido complexes, **2** and **4**. These results suggest the molecular structures of each pair of Os^II^ iminopyridine complexes are mirror images of each other.

To probe the formation of both diastereomers differing at the metal configuration, the first fraction of crystals of **3** was filtered off and the remaining solution was concentrated under reduced pressure to give an orange crystalline product. The ^1^H NMR data for the orange product showed a diastereomeric mixture of (*R*_Os_,*R*_C_)- and (*S*_Os_,*R*_C_)*-*[Os(η^6^-*p*-cym)(ImpyMe)Cl]PF_6_ in approximately 1:1 ratio instead of a single diastereomer (see the Supporting Information, Figure S1). This finding is consistent with a previous report on Ru^II^ and Os^II^ arene salicylaldiminates complexes.^29^ There were only small differences between the ^1^H NMR chemical shifts of both diastereomers, a fact that made it difficult to identify which was the most stable product. For this reason, an initial crystallization step was necessary to obtain pure chiral-at-Os^II^ complexes that separately exhibit different chemical shifts.

**Figure 2:**
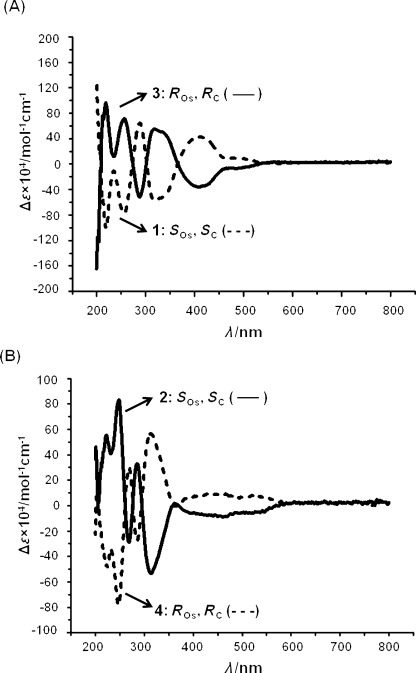
Circular dichroism spectra for the two pairs of Os^II^ arene iminopyridine complexes: (A) 1 and 3; (B) 2 and 4.

**Circular dichroism**: To gain further understanding of the enantiomeric relationships between these pairs of complexes, CD spectra were recorded. This technique measures the differential absorption of left- and right circularly polarized light and is widely used to confirm chiral purity. The CD spectra were recorded for each of the four Os^II^ iminopyridine complexes in methanol. The chlorido complexes, **1** and **3**, and iodido complexes, **2** and **4**, gave complementary CD spectra (Figure 2). Complex **1** showed positive Cotton effects at 412 and 288 nm, and negative Cotton effects at 318, 256 and 220 nm. Complex **2** showed positive Cotton effects at 285, 247 and 221 nm, and negative Cotton effects at 450, 313 and 268 nm. Opposite Cotton effects were observed for complexes **1** and **2** and for complexes **3** and **4**. Although CD cannot give information on the absolute configuration at the chiral osmium center in the individual complexes in solution, such results confirm that the two molecular structures within the two pairs of Os^II^ iminopyridine complexes are mirror images.^49^

**Stability in aqueous solution**: Although chiral metal centers are usually stable in the solid state at ambient and physiological temperatures, they can behave differently in solutions in which epimerization can occur at ambient temperature with a half-life (*τ*_1/2_) less than 24 h.^50^ The configurational stability of the metal center in organometallic complexes in solution may depend on the ligands around the metal;^51^–^53^ for example, early work on the Ru^II^ arene iodido complexes [Ru(η^6^-*p*-cym)(LL*)I] (LL*=(S_C_)-(−)-dimethyl(1-phenylethyl)amine) showed that they were more configurationally labile compared with their chlorido analogues.^54^ However, there are no reports on the analogous Os^II^ arene complexes. In general, epimerization occurs in solvents such as acetone, methanol, CH_2_Cl_2_, or CHCl_3_ giving rise to the thermodynamic product as the major epimer in solution. Nevertheless, diastereomeric complexes have been shown to be stereochemically stable in hydrocarbon-based solvents.^55^

The configurational stability of Os^II^ arene complexes that are designed as anticancer agents is of particular interest. Each of the four Os^II^ arene complexes were dissolved in 10 % CD_3_OD/90 % D_2_O phosphate buffer (pH* 7.4), and ^1^H NMR spectra were recorded before and after incubation for 24 h at 310 K. The two iodido complexes, **2** and **4**, showed good stability with no change in the ^1^H NMR spectra after the incubation period. In contrast, the ^1^H NMR spectra for both chlorido complexes, **1** and **3**, showed new peaks, which may correspond to epimerization or aquation products (see the Supporting Information, Figure S2). When complexes **1** and **3** were incubated at 310 K in the presence of a high molar excess (1000-fold) of sodium chloride (to suppress aquation) and the ^1^H NMR spectra recorded, no new peaks appeared indicating that the aforementioned new resonances correspond to aquation products and that these complexes are stable towards epimerization. The possibility of substitution of iodide by chloride was investigated for the two iodido complexes, **2** and **4**. The NMR data showed no substitution of iodide by chloride for either **2** or **4** (100 μM) at high concentrations of Cl^−^ (5000 mol equiv, 500 mM) (see the Supporting Information, Figure S2).

**Anticancer activity**: Iminopyridine- and azopyridine-containing complexes^56^, ^57^ with Ru and Os centers can exhibit potent anticancer activity.^58^–^62^ In particular, an Os^II^ arene azopyridine complex is active in vivo.^56^ Therefore, the anticancer activities of the four Os^II^ arene iminopyridine complexes **1**–**4** were studied in the human ovarian cancer cell line A2780. After 24 h incubation followed by 72 h recovery time, the two chlorido Os complexes, **1** and **3**, showed moderate anticancer activity, with IC_50_ values of approximately 20 μm, similar to the value obtained for the mixture of (*R*_Os_,*R*_C_)- and (*S*_Os_,*R*_C_)*-*[Os(η^6^-*p*-cym)(ImpyMe)Cl]PF_6_ (Table 4) and significantly higher than that of cisplatin (2 μM). On the other hand, the IC_50_ values of the two iodido osmium complexes, **2** and **4**, are in the same range as that of cisplatin (Table 4). These two iodido complexes were further screened in the NCI (National Cancer Institute) panel of 60 human tumor cell lines at five concentrations^63^, ^64^ and both **2** and **4** showed potent anticancer activities with mean IC_50_ values of 9.55 and 7.58 μM, respectively. In contrast, the two chlorido complexes, **1** and **3**, were not sufficiently active when tested against the NCI 60-cell-line panel (mean IC_50_ values >10 μM) to warrant 5-dose testing. The mean growth inhibition parameters determined in the NCI screen of MG-MID (full-panel mean-graph midpoint) values of IC_50_ (the concentration that inhibits cell growth by 50 %), TGI (the concentration that inhibits cell growth by 100 %), and LC_50_ (the concentration that kills 50 % of the original cells) are listed in Table 5. The details for each cell line and the values of IC_50_, TGI, and LC_50_ are shown in Table S1 (see the Supporting Information). Similar to cisplatin, the two Os^II^ iodido complexes showed a broad range of anticancer activities towards different cell lines, with IC_50_ values ranging from nanomolar to micromolar (530 nM to >100 μM).

**Table 4:** IC_50_ values for the A2780 ovarian cancer cell line.

Complex	IC_50_ [μM]
(*S*_Os_,*S*_C_)*-*[Os(η^6^-*p*-cym)(ImpyMe)Cl]PF_6_ (**1**)	22.3 (±1.6)
(*S*_Os_,*S*_C_)*-*[Os(η^6^-*p*-cym)(ImpyMe)I]PF_6_ (**2**)	1.9 (±0.2)
(*R*_Os_,*R*_C_)*-*[Os(η^6^-*p*-cym)(ImpyMe)Cl]PF_6_ (**3**)	18.3 (±1.7)
(*R*_Os_,*R*_C_)*-*[Os(η^6^-*p*-cym)(ImpyMe)I]PF_6_ (**4**)	0.60 (±0.02)
(*R*_Os_,*R*_C_) and (*S*_Os_,*R*_C_)*-*[Os(η^6^-*p*-cym)(ImpyMe)Cl]PF_6_ mixture[a]	19.0 (±1.1)
cisplatin	2.0 (±0.2)

[a] Ratio approximately 1:1.

**Table 5:** Mean IC_50_, TGI and LC_50_ values from the NCI-60 data for complexes 2 and 4.

Complex[a]	IC_50_ [μM][b]	TGI [μM][c]	LC_50_ [μM][d]
(*S*_Os_,*S*_C_)*-*[Os(η^6^-*p*-cym)(ImpyMe)I]PF_6_ (**2**)	9.55	61.7	91.2
(*R*_Os_,*R*_C_)*-*[Os(η^6^-*p*-cym)(ImpyMe)I]PF_6_ (**4**)	7.58	53.7	89.1
cisplatin[e]	1.49	9.33	44.0

[a] NCI codes for complex **2**: NSC: D-758116/1 and **4**: NSC: D-758118/1.

[b] IC_50_=the concentration that inhibits cell growth by 50 %.

[c] TGI=the concentration that inhibits cell growth by 100 %.

[d] LC_50_=the concentration that kills 50 % of the original cells.

[e] Cisplatin data from NCI/DTP screening: March 2012, 48 h incubation time.^79^

**Catalysis of imine reduction**: Organometallic ruthenium,^40^ rhodium^65^ and iridium^66^ complexes have been found to act as transfer-hydrogenation catalysts for the asymmetric reduction of ketones and imines. However, there are very few reports of the catalytic activity of osmium complexes,^43^, ^44^ especially for Os^II^ arene complexes.^67^ The possible catalytic activity of the two Os^II^ arene iminopyridine iodido complexes, **2** and **4**, for cyclic imine (6,7-dimethoxy-1-methyl-3,4-dihydroisoquinoline) reduction was therefore evaluated. We observed a reasonable conversion (20–76 %) and low *ee* value (20–22 %) for reactions with both Os^II^ complexes in less than 24 h (see the Supporting Information, Figure S4),^68^ demonstrating potential of the complexes as transfer-hydrogenation catalysts. This appears to be the first report of this type of Os^II^ arene iminopyridine iodido complex acting as a catalyst in transfer hydrogenation. Although the asymmetric reduction of imines by Ru^II^ complexes has been studied in some detail, the mechanism of the reaction, in contrast to ketone reduction, is unclear. A recent report suggests that the reduction takes place through an “open” transition state in which binding to the N–H of the complex (typical for ketone reduction) is not required. This is supported by experiments conducted on stoichiometric reduction systems and also by molecular modeling. A similar mode of reduction has been proposed for enzyme-bound imine reductions.^69^, ^70^ The proposed mechanism retains the CH–π interaction that has been proposed for similar systems, although it is not clear whether this is operating in the current system.

## Discussion

**Crystal structures**: Only one chiral configuration at the Os^II^ center was observed in the X-ray crystal structures of chiral-at-Os^II^ iminopyridine complexes **1**, **2**, **3**, and **4**, a fact that is consistent with the indication from the ^1^H NMR data. Thus, the use of two enantiomeric ligands in this work for the crystallization of pure diastereomers allowed us to successfully isolate two pairs of enantiomers (**1**, **3** and **2**, **4**, respectively). These results differ markedly from our previous findings on the Os^II^ iminopyridine complex [Os(η^6^-*p*-cym)(Impy-OH)I]PF_6_, whose X-ray crystal structure showed a racemic mixture of enantiomers, (*S*_Os_)-[Os(η^6^-*p*-cym)(Impy-OH)I]PF_6_ and (*R*_Os_)-[Os(η^6^-*p*-cym)(Impy-OH)I]PF_6_.^14^ Chiral resolution of the second set of enantiomers in solution was hampered by their low solubility.

The assignment of chirality for the two pairs of osmium diastereomers **1**/**3** and **2**/**4** is consistent with their complementary CD spectra (Figure 2). It is apparent that the absolute stereochemical arrangements of the ligands around the osmium center in the complexes with the same chelating ligand **1** and **2** (chelating ligand=(*S*)-ImpyMe), **3** and **4** (chelating ligand=(*R*)-ImpyMe) are similar. These complexes do not exhibit the intramolecular “β-phenyl effect” found in previously reported organometallic arene complexes derived from similar chiral ligands. This stabilizing effect consists of an edge-to-face CH–π attractive interaction between the arene hydrogen atoms and a phenyl group from the optical active ligand, consequently giving rise to the thermodynamic product. Most of the published examples of diastereomeric Ru^II^ and Os^II^ arene complexes showing the “β-phenyl effect” are neutral complexes^71^, ^72^ or contain different counterions (for example, ClO_4_^−^, Cl^−^, PF_6_^−^).^72^

The crystal structures of compounds **1**–**4** show the phenyl substituent from the ImpyMe ligand orientated downwards in order to avoid steric interactions with the *p*-cymene. A structural difference found between the chlorido and iodido complexes is the spatial arrangement of the methyl substituent from the ImpyMe ligand with respect to the monodentate ligand. The methyl group is directed towards the halide ligand in the chlorido complexes, **1** and **3**, but it points in the opposite direction in the iodido analogues, **2** and **4**, probably owing to steric repulsion by the bulky coordinated iodide. It is notable that in the chlorido complexes, an intermolecular non-classical *C–H⋅⋅⋅F bond interaction between the PF_6_^−^ counterion and the hydrogen atom attached to the chiral carbon atom of the iminopyridine ligand is observed, whereas in the case of the iodido analogues this interaction occurs with the imine hydrogen. Additionally the PF_6_^−^ anion hydrogen bonds to the *p*-cymene ligand and the pyridine ring of the ImpyMe ligand (Tables 1 and 2).

Regarding the thermodynamic/kinetic origin of the crystallized products, equilibration between the diastereomers at ambient temperature can be a fast process in which the thermodynamic product is obtained as the major epimer in solution.^51^ The equilibrium constant and configurational lability at the metal center depend on the temperature and the solvent, and some ruthenium arene complexes have a stable metal configuration even at high temperatures.^26^, ^55^ Several ^1^H NMR kinetic studies carried out by Brunner et al. on configurationally labile Ru^II^ and Os^II^ half-sandwich diastereomers concluded that at low temperatures (193–195 K)^29^, ^73^ the major epimer (thermodynamic product) exists as the only compound in solution. An increase in temperature (223–294 K) favors the formation of the kinetic diastereomer, the percentage of which is higher at higher temperatures. Frequently the crystallization of these diastereomeric mixtures in solution gives rise to single crystals that contain only the less soluble diastereomer.

Taking all this into account, complexes **1**, **2**, **3**, and **4** can be considered as the kinetic products of the reaction as well as the less soluble epimers at the temperature at which their crystallization from methanol took place (277 K). Few examples of Os^II^ half-sandwich complexes crystallized as diastereomerically pure compounds have been reported.^29^, ^67^, ^73^

**Circular dichroism**: The CD spectra of the pairs of enantiomers (*S*_Os_,*S*_C_)*-*[Os(η^6^-*p*-cym)(ImpyMe)Cl]PF_6_ (**1**) and (*R*_Os_,*R*_C_)*-*[Os(η^6^-*p*-cym)(ImpyMe)Cl]PF_6_ (**3**), (*S*_Os_,*S*_C_)*-*[Os(η^6^-*p*-cym)(ImpyMe)I]PF_6_ (**2**) and (*R*_Os_,*R*_C_)*-*[Os(η^6^-*p*-cym)(ImpyMe)I]PF_6_ (**4**) showed several absorption bands with maximum intensities in the range 220–350 nm. A shoulder around 340 nm is also observed in spectra of the chlorido compounds, **1** and **3**. These bands may be attributable to π→π* and n→π* transitions of the coordinated chiral iminopyridine ligand. Additionally, both pairs of enantiomers displayed two broad bands of lower intensity between 360–550 nm due to metal-based transitions. The opposite Cotton effects observed for **1** and **2** compared to **3** and **4**, respectively, Figure 2, indicate that they are two pairs of mirror-image complexes.

**Aqueous configuration stability**: There was no epimerization at the Os^II^ center during a 24 h incubation at 310 K of both the chlorido, **1** and **3**, and iodido, **2** and **4**, diastereomeric Os^II^ iminopyridine arene complexes under biologically relevant conditions. This suggests that they are stable enough to allow further investigations of the effects of chirality of the osmium metal center on biological activity. As aquation and nucleophilic substitution of the metal–halide bond are involved in the general mechanism associated with DNA binding for Ru^II^ and Os^II^ arene anticancer complexes,^74^ the mechanism of anticancer activity of these inert Os^II^ arene iminopyridine iodido complexes is unlikely to involve DNA as a target in contrast to some previously reported Os^II^ arene complexes.^74^

**Density functional theory calculations**: It is apparent that the synthetic route used for all the four osmium arene iminopyridine halido complexes can give rise to both *R* and *S* configurations at Os. To compare the thermodynamic stabilities of the diastereomers, density functional theory (DFT) calculations were performed to optimize the geometry and calculate energies. These calculations were run at the B3LYP/LANL2DZ/6-31G** level in gas phase by using Gaussian 03.

For the two osmium chlorido complexes, the sequence of thermodynamic stability was: **3** (*R*_Os_,*R*_C_)>**1** (*S*_Os_,*S*_C_)>(*S*_Os_,*R*_C_) = (*R*_Os_,*S*_C_) configuration. According to computational results, the **1** configuration is only 0.043 kJ mol^−1^ less stable than **3**, whereas the (*R*_Os_,*S*_C_) and (*S*_Os_,*R*_C_) configurations are similar and 6.93 kJ mol^−1^ less stable than **1**. For the two osmium iodido complexes, the sequence of thermodynamic stability follows the order: (*S*_Os_,*R*_C_) configuration>(*R*_Os_,*S*_C_) configuration>**2** (*S*_Os_,*S*_C_)=**4** (*R*_Os_,*R*_C_). According to the calculated energies, the (*R*_Os_,*S*_C_) configuration is only 0.002 kJ mol^−1^ less stable than the (*S*_Os_,*R*_C_) configuration, whereas **2** and **4** are 10.06 kJ mol^−1^ less stable than the (*R*_Os_,*S*_C_) configuration. Although the differences in energy are small, the computational results indicate that **1** and **3** are the thermodynamically favored isomers, while **2** and **4** are not thermodynamically favored isomers in terms of the chirality at osmium. Their separation may arise from solubility differences between the epimers.

**Figure 3:**
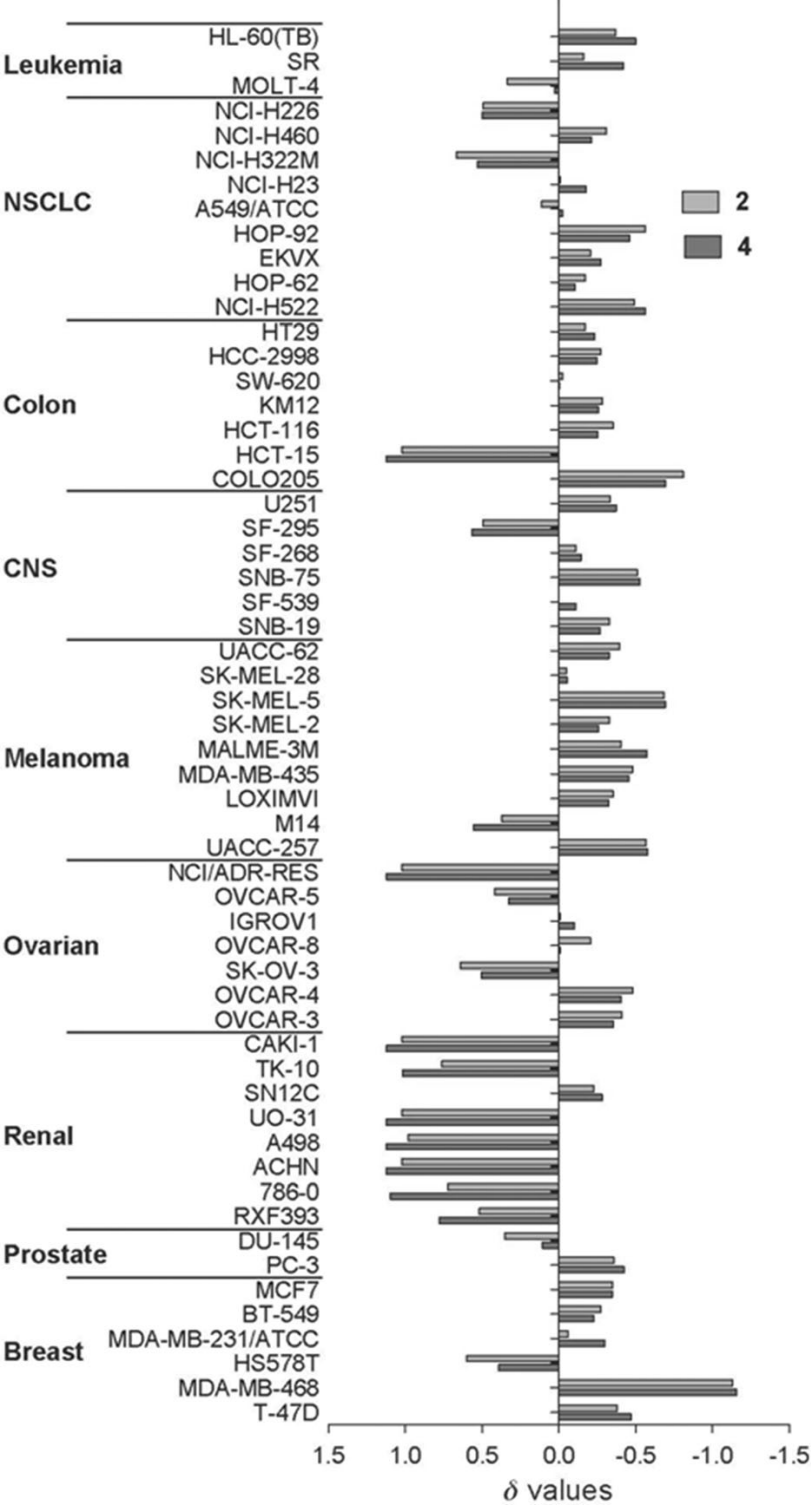
Overlay of mean graph for Os^II^ arene iodido iminopyridine complexes 2 and 4 based on IC_50_ values from 60-cell-line screening (57 actual) by the National Cancer Institute Developmental Therapeutics Program. The average line represents the mean IC_50_ values for these compounds; bars pointing to the right indicate higher activity and to the left indicate lower activity compared with the mean value. NSCLC = non-small cell lung cancer; CNS = central nervous system.

**Anticancer activity**: Further analysis of the in vitro anticancer efficacy of complexes **2** and **4** by the NCI revealed a broad spectrum of activity with promising selectivity towards melanoma and breast cancer cell lines. The breast cell line MDA-MB-468 showed particularly promising results with IC_50_ values in the sub-micromolar range (703 nM for **2** and 530 nM for **4**). Also notable is the low selectivity of **2** and **4** for renal cancer cell lines (Figure 3), with IC_50_>100 μM for most of the renal cell lines in the NCI-60 screening.

The COMPARE algorithm is an open tool developed by the NCI to quantify directly the similarity in cell line sensitivity between compounds.^64^ Each NCI-60 mean graph is taken as a fingerprint for the compound and is quantitatively compared to mean graphs for other compounds, producing a Pearson’s correlation coefficient (PCC) between −1 and 1 as a similarity measure. This method has been used successfully to predict mechanisms of action of emerging drugs by highlighting similarity in mean graph fingerprints to drugs of known mechanism in the NCI databases.^75^

A single analysis using the COMPARE algorithm for each complex against the NCI/DTP (Developmental Therapeutics Program) Standard Agents Database, housing 171 known anticancer compounds, was conducted.^76^ The results provide preliminary indications of a possible mechanism of action based on a correlation of the NCI 60-cell-line patterns of sensitivity.^77^ The three endpoints (IC_50_, TGI, and LC_50_) were used in the algorithm and those agents in the database with the highest PCC values were analyzed (Tables S2–S4). The highest PCC value for each endpoint for both complexes **2** and **4** was for vinblastine sulfate, an inhibitor of tubulin polymerization. The quantitative analysis of the selectivity patterns between complexes **2** and **4** gave PCC values of 0.973 (IC_50_), 0.969 (TGI), and 0.976 (LC_50_) for different endpoints, indicating that **2** and **4** may share high similarity in their mechanisms. This is demonstrated in the mean graphs in Figure 3, where the pattern of sensitivity is almost identical. We assessed whether cisplatin showed a significant correlation to either **2** or **4**, and found that this comparison produced a PCC value of −0.298. This negative value highlights that cisplatin has a distinctly different pattern of selectivity and suggests a different mechanism of action from complexes **2** and **4** (Table 6).

**Table 6:** COMPARE analysis showing the highest correlations for complexes 2 and 4 with known anticancer drugs, as indicated by their PCC (Pearson′s correlation coefficient) values.

Complex	PCC	Name	Mechanism
(*S*_Os_,*S*_C_)*-*[Os(η^6^-*p*-cym)(ImpyMe)I]PF_6_ (**2**)	0.743	vinblastine sulfate	antimicrotubule agent
(*R*_Os_,*R*_C_)*-*[Os(η^6^-*p*-cym)(ImpyMe)I]PF_6_ (**4**)	0.754	vinblastine sulfate	antimicrotubule agent

The prevention of polymerization of microtubules in vitro was investigated by assessing whether Os^II^ complexes **2** and **4** interact directly with tubulin. Relative concentrations of Os^II^ complexes (10 μM) were selected according to the IC_50_ values from the cell tests. Purified, unpolymerized tubulin was incubated with Os^II^ compounds and the polymerization process monitored at 310 K for 60 min. Taxol and colchicines were used as positive controls for polymerization facilitation and inhibition, respectively.^78^ Under these conditions, no inhibition of polymerization by Os^II^ complexes was observed (see the Supporting Information, Figure S3), a fact that indicated that the mechanism of action does not involve direct interaction with tubulin.

## Conclusion

There are few reported studies of isolated organometallic complexes with chirally pure Os^II^ centers. In this work, through incorporation of an additional chiral center into a chelated iminopyridine ligand, we have separated four chiral Os^II^ arene anticancer complexes by fractional crystallization: two iodido complexes, (*S*_Os_,*S*_C_)-[Os(η^6^-*p*-cym)(ImpyMe)I]PF_6_ (**2**, containing (*S*)-ImpyMe: *N*-(2-pyridylmethylene)-(*S*)-1-phenylethylamine), and (*R*_Os_,*R*_C_)-[Os(η^6^-*p*-cym)(ImpyMe)I]PF_6_ (**4**, containing (*R*)-ImpyMe: *N*-(2-pyridylmethylene)-(*R*)-1-phenylethylamine), and the two chlorido derivatives (*S*_Os_,*S*_C_)*-*[Os(η^6^-*p*-cym)(ImpyMe)Cl]PF_6_ (**1**) and (*R*_Os_,*R*_C_)-[Os(η^6^-*p*-cym)(ImpyMe)Cl]PF_6_ (**3**). Their X-ray crystal structures and CD spectra verified their mirror image configurations. Interestingly, the two iodido Os^II^ arene complexes, **2** and **4**, showed more promising anticancer activity against A2780 human ovarian cancer cell line and the NCI 60-cell-line screening compared with the two chlorido Os^II^ arene complexes, **1** and **3**. Quantitative analysis of the NCI 60-cell-line screen using the COMPARE algorithm showed that the two potent iodido complexes have surprisingly similar selectivity patterns to one another and to an anti-microtubule drug, vinblastine sulfate. However, no direct effect towards tubulin polymerization was found for **2** and **4**, a fact that may indicate a different or indirect mechanism of action. Other than anticancer activity, **2** and **4** also demonstrated potential as hydrogenation transfer catalysts for imine reduction.

## Experimental Section

**Starting materials**: OsCl_3_**⋅**3H_2_O was purchased from Alfa-Aesar. Ethanol and methanol were dried over Mg/I_2_ or anhydrous quality was used (Aldrich). All other reagents were obtained from commercial suppliers and used as received. The preparations of the starting materials [{Os(η^6^-*p*-cym)Cl_2_}_2_] and [{Os(η^6^-*p*-cym)I_2_}_2_] have been previously reported.^57^ The synthesis and purification of the chiral pure iminopyridine ligands, (*S*)-ImpyMe: *N*-(2-pyridylmethylene)-(*S*)-1-phenylethylamine, (*R*)-ImpyMe: *N*-(2-pyridylmethylene)-(*R*)-1-phenylethylamine, were carried out according to the literature method.^45^ The A2780 human ovarian carcinoma cell line was purchased from European Collection of Animal Cell Cultures (Salisbury, UK), RPMI-1640 media and trypsin were purchased from Invitrogen, bovine serum from Biosera, penicillin, streptomycin, trichloroacetic acid (TCA), and sulforhodamine B (SRB) from Sigma–Aldrich, and tris(hydroxymethyl)aminomethane from Formedium.

**Syntheses and characterization**: All the Os^II^ compounds were synthesized and crystallized by a general method that is described for compound **1**. Single crystals suitable for X-ray diffraction were obtained for all these compounds and were used for all the chemical and biological tests.

**(*S***_**Os**_,***S***_**C**_**)***-***[Os(η^6^-*p*-cym)(ImpyMe)Cl]PF_6_ (1)**: [{Os(η^6^-*p*-cym)Cl_2_}_2_] (50.0 mg, 0.063 mmol) was dissolved in methanol (20 mL) at 313 K. (*S*)-ImpyMe (26.9 mg, 0.12 mmol) in methanol (10 mL) was added drop-wise. The solution changed color from orange to red immediately, and was stirred at ambient temperature (ca. 293 K) for 2 h. Ammonium hexafluorophosphate (41.2 mg, 0.24 mmol) was added. Then the solution was left in a fridge at 277 K for 24 h. Dark brown colored single crystals were obtained and were collected by filtration, washed with cold ethanol and diethyl ether, and dried under vacuum. Yield (single crystals): 54.0 mg (58.7 %); ^1^H NMR (400 MHz, (CD_3_)_2_CO): *δ*= 9.59 (d, *J*=6 Hz, 1 H), 9.32 (s, 1 H), 8.47 (d, *J*=8 Hz, 1 H), 8.32 (t, *J*=6 Hz, 1 H), 7.73 (t, *J*=6 Hz, 2 H), 7.62–7.55 (m, 3 H), 6.55 (d, *J*=6 Hz, 1 H), 6.16 (d, *J*=6 Hz, 1 H), 6.06 (qd, *J*=6, 8 Hz, 1 H), 5.89 (d, *J*=6 Hz, 2 H), 2.65 (s, 3 H), 2.54–2.42 (m, 1 H), 2.38 (s, 6 H), 1.93 (d, *J*=6 Hz, 3 H), 1.11 (d, *J* = 7 Hz, 3 H), 0.89 ppm (d, *J* = 7 Hz, 3 H); ESI-MS: *m*/*z* calcd for C_24_H_28_ClN_2_Os: 571.2 [*M*]^+^; found 571.1; elemental analysis calcd (%) for C_24_H_28_ClF_6_N_2_OsP: C 40.31, H 3.95, N 3.92; found: C 40.30, H 3.87, N 3.90; CD (CH_3_OH): *λ*_max_ (Δ*ε*): 412 (+42), 318 (−58), 288 (+64), 256 (−78), 220 nm (−100 mol^−1^ cm^−1^). The brown single crystals of **1** were suitable for study by X-ray diffraction.

**(*S***_**Os**_,***S***_**C**_**)***-***[Os(η^6^-*p*-cym)(ImpyMe)I]PF_6_ (2)**: Prepared by following the same method as for complex **1**. Yield (single crystals): 28.9 mg (33.8 %); ^1^H NMR (400 MHz, (CD_3_)_2_CO): *δ*= 9.57 (d, *J*=6 Hz, 1 H), 9.43 (s, 1 H), 8.48 (d, *J*=8 Hz, 1 H), 8.25 (t, *J*=6 Hz, 1 H), 7.77 (t, *J*=6 Hz, 2 H), 7.52–7.48 (m, 3 H), 6.49 (d, *J*=6 Hz, 1 H), 6.26 (d, *J*=6 Hz, 1 H), 6.08 (qd, *J*=6, 8 Hz, 1 H), 6.04 (d, *J*=6 Hz, 1 H), 5.83 (d, *J*=6 Hz, 1 H), 2.84 (s, 3 H), 2.67–2.60 (m, 1 H), 2.65 (s, 6 H), 2.13 (d, *J*=7 Hz, 3 H), 1.10 (d, *J* = 7 Hz, 3 H), 0.91 ppm (d, *J* = 7 Hz, 3 H); ESI-MS: *m*/*z* calcd for C_24_H_28_IN_2_Os: 663.1 [*M*]^+^; found 663.0. elemental analysis calcd (%) for C_24_H_28_F_6_IN_2_OsP: C 35.74, H 3.50, N 3.47; found: C 35.76, H 3.53, N 3.36; CD (CH_3_OH): *λ*_max_ (Δ*ε*): 450 (−9), 313 (−57), 285 (+27), 268 (−30), 247 (+82), 221 nm (+51 mol^−1^ cm^−1^). The brown single crystals grown from a cooled methanol solution (277 K) were suitable for X-ray diffraction studies.

**(*R***_**Os**_,***R***_**C**_**)**-**[Os(η^6^-*p*-cym)(ImpyMe)Cl]PF_6_ (3)**: Prepared by following the same method as for complex **1**. Yield (single crystals): 25.5 mg (27.7 %); ^1^H NMR (400 MHz, (CD_3_)_2_CO): *δ* = 9.59 (d, *J*=6 Hz, 1 H), 9.32 (s, 1 H), 8.47 (d, *J*=8 Hz, 1 H), 8.32 (t, *J*=6 Hz, 1 H), 7.73 (t, *J*=6 Hz, 2 H), 7.62–7.55 (m, 3 H), 6.55 (d, *J*=6 Hz, 1 H), 6.16 (d, *J*=6 Hz, 1 H), 6.06 (qd, *J*=6, 8 Hz, 1 H), 5.89 (d, *J*=6 Hz, 2 H), 2.65 (s, 3 H), 2.54–2.42 (m, 1 H), 2.38 (s, 6 H), 1.93 (d, *J*=6 Hz, 3 H), 1.11 (d, *J* = 7 Hz, 3 H), 0.89 ppm (d, *J* = 7 Hz, 3 H); ESI-MS: *m*/*z* calcd for C_24_H_28_ClN_2_Os: 571.2 [*M*]^+^; found 571.1; elemental analysis calcd (%) for C_24_H_28_ClF_6_N_2_OsP: C 40.31, H 3.95, N 3.92; found: C 40.16, H 3.80, N 3.90;. CD (CH_3_OH): *λ*_max_ (Δ*ε*): 412 (−41), 318 (+58), 288 (−62), 256 (+78), 220 nm (+99 mol^−1^ cm^−1^). Orange single crystals suitable for X-ray crystallography were obtained after cooling a portion of the methanol solution at 277 K.

**(*R***_**Os**_,***R***_**C**_**)-[Os(η^6^-*p*-cym)(ImpyMe)I]PF_6_ (4)**: Prepared by following the same method as for complex **1**. Yield (single crystals): 25.2 mg (35.6 %); ^1^H NMR (400 MHz, (CD_3_)_2_CO): *δ*= 9.57 (d, *J*=6 Hz, 1 H), 9.43 (s, 1 H), 8.48 (d, *J*=8 Hz, 1 H), 8.25 (t, *J*=6 Hz, 1 H), 7.77 (t, *J*=6 Hz, 2 H), 7.52–7.48 (m, 3 H), 6.49 (d, *J*=6 Hz, 1 H), 6.26 (d, *J*=6 Hz, 1 H), 6.08 (qd, *J*=6, 8 Hz, 1 H), 6.04 (d, *J*=6 Hz, 1 H), 5.83 (d, *J*=6 Hz, 1 H), 2.84 (s, 3 H), 2.67–2.60 (m, 1 H), 2.65 (s, 6 H), 2.13 (d, *J*=7 Hz, 3 H), 1.10 (d, *J* = 7 Hz, 3 H), 0.91 ppm (d, *J* = 7 Hz, 3 H); ESI-MS: *m*/*z* calcd for C_24_H_28_IN_2_Os: 663.1 [*M*]^+^; found 663.0; elemental analysis calcd (%) for C_24_H_28_F_6_IN_2_OsP: C 35.74, H 3.50, N 3.47; found: C 35.63, H 3.47, N 3.33; CD (CH_3_OH): *λ*_max_ (Δ*ε*): 450 (+9), 313 (+56), 285 (−27), 268 (+30), 247 (−78), 222 nm (−47 mol^−1^ cm^−1^). Dark brown single crystals suitable for X-ray crystallography were obtained after cooling a portion of the methanol solution at 277 K.

**Instrumentation**

*NMR spectroscopy*: ^1^H NMR spectra were acquired in 5 mm NMR tubes at 298 K on either Bruker DPX-400, Bruker DRX-500, or Bruker AV II 700 spectrometers. ^1^H NMR chemical shifts were referenced to [D_6_]acetone (2.09 ppm). All data processing was carried out using MestReC or TOPSPIN version 2.0 (Bruker U.K. Ltd.).

*Electrospray ionisation mass spectrometry (ESI-MS)*: Spectra were obtained by preparing the samples in 50 % CH_3_CN and 50 % H_2_O (v/v) and infusing into the mass spectrometer (Varian 4000). The mass spectra were recorded with a scan range of *m*/*z* 500–1000 for positive ions.

*Elemental analysis*: Elemental analysis (carbon, hydrogen, and nitrogen) was carried out through Warwick Analytical Service using an Exeter analytical elemental analyzer (CE440).

*pH* measurements*: pH* (pH meter reading from D_2_O solution without correction for effects of deuterium on glass electrode) values were measured at ambient temperature before the NMR spectra were recorded, using a Corning 240 pH meter equipped with a micro-combination electrode calibrated with Aldrich buffer solutions at pH 4, 7, and 10.

*X-ray crystallography*: X-ray diffraction data for **1**, **2**, **3**, and **4** were obtained on an Oxford Diffraction Gemini four-circle system with a Ruby CCD area detector using Mo_Kα_ radiation.^80^ Absorption corrections were applied by using ABSPACK. The crystals were mounted in oil and held at 100(2) K with the Oxford Cryosystem Cryostream Cobra. The structures were solved by direct methods using SHELXS (TREF) with additional light atoms found by Fourier methods^81^ and refined against *F*^2^ using SHELXL 97.^82^ Hydrogen atoms were added at calculated positions and refined using a riding model with freely rotating methyl groups. Anisotropic displacement parameters were used for all non-H atoms; H atoms were given isotropic displacement parameters equal to 1.2 (or 1.5 for methyl hydrogen atoms) times the equivalent isotropic displacement parameter of the atom to which the H atom is attached. The graphics for the crystal structures were made with Mercury 2.4. CCDC-941759 http://www.ccdc.cam.ac.uk/cgi-bin/catreq.cgi(**1**), 941760 http://www.ccdc.cam.ac.uk/cgi-bin/catreq.cgi(**2**), 941761 http://www.ccdc.cam.ac.uk/cgi-bin/catreq.cgi(**3**), and 941762 http://www.ccdc.cam.ac.uk/cgi-bin/catreq.cgi(**4**) contain the supplementary crystallographic data for this paper. These data can be obtained free of charge from The Cambridge Crystallographic Data Centre via www.ccdc.cam.ac.uk/data_request/cif.

*Cell cultures*: A2780 human ovarian cancer cell lines (ECACC, Salisbury, UK) were cultured in RPMI 1640 cell culture medium supplemented with 1 mM sodium pyruvate, 2 mm l-glutamine, and 10 % fetal bovine serum.

*Circular dichroism*: Circular dichroism spectra of **1**, **2**, **3**, and **4** were recorded on a J-815 circular dichroism spectropolarimetrer (Jasco, UK) at ambient temperature (ca. 293 K). The spectra were determined for all the samples at a concentration of 1 mM in methanol using a quartz cuvette of 0.1 cm path length, scan speed of 200 nm min^−1^, 1 nm band width, 0.2 nm data pitch, and 0.5 s of response time.

**Methods**

*Aquation*: Solutions of **1**, **2**, **3**, or **4** in 10 % CD_3_OD/90 % D_2_O phosphate buffer (v/v) were prepared by dissolution in CD_3_OD followed by a dilution with D_2_O phosphate buffer (pH*=7.2). NMR spectra were recorded after 24 h of incubation at 310 K. The extent of aquation was determined from ^1^H NMR peak integrals.

*Determination of A2780 IC_50_ values*: The method was similar to that previously described.^57^

*NCI 60-cell-line screen*: The cells were treated for 48 h at five concentrations ranging from 0.01 to 100 μM. Three endpoints were calculated: IC_50_ (the concentration that inhibits cell growth by 50 %); TGI (the concentration that inhibits cell growth by 100 %); LC_50_ (the concentration that kills 50 % of the original cells); MG-MID (full-panel mean-graph midpoint). Cisplatin data were from NCI/DTP screening: Oct 2009, 48 h incubation.

*Data evaluation, mean graph analysis, and COMPARE analysis*: Anti-proliferative efficacies of test compounds in both assays were described by inhibitory concentrations (IC_50_ values), reflecting concentration-dependent cytotoxicity. Complexes **2** and **4** were selected for study in the human tumor 60-cell-line panel of the Developmental Therapeutics Program (DTP) of the National Cancer Institute (NCI), a panel that includes nine tumor-type subpanels. The cells were treated for 48 h at five concentrations ranging from 0.01 to 100 μM. Three endpoints were calculated: IC_50_ (the concentration that inhibits cell growth by 50 %); TGI (the concentration that inhibits cell growth by 100 %); LC_50_ (the concentration that kills 50 % of the original cells); MG-MID (full-panel mean-graph midpoint). The MG-MID value therefore represents an antiproliferative fingerprint of a compound. The COMPARE analysis was carried out using the NCI/DTP Standard Agents database, a collection of 171 known anticancer compounds, to provide preliminary indications on a possible mechanism of action based on data obtained in a panel of tumor cell lines tested in the 60-cell-line screen in vitro.^77^ Compounds that have a high correlation coefficient with known drugs have generally been found to have similar mechanisms of action. High correlation (*ρ* ≥ 0.6) to a specific standard agent indicated a similar anticancer mechanism of action. Low correlations to all standard agents indicated a novel anticancer mechanism of action not represented by the standard agent database.

*Tubulin polymerization assay*: A cytoskeleton tubulin polymerization assay kit (catalog no. BK004) was used in the tubulin polymerization study. Briefly, 10 μL of general tubulin buffer (80 mM PIPES, pH 6.9, 2 mM MgCl_2_, and 0.5 mM EGTA) containing osmium compound, colchicine, or taxol was pipetted into the pre-warmed 96-well microplate. Tubulin (defrosted to room temperature from 213 K and then placed on ice before use) was diluted with tubulin polymerization buffer with 1 mM GTP to a final concentration of 4 mg mL^−1^. Diluted tubulin (100 μL) was added into the wells containing **2**, **4**, colchicine, or taxol. Diluted tubulin (100 μL) mixed with general tubulin buffer (10 μL) served as control. The absorbance at 340 nm was read immediately with a Tecan microplate reader.

*Catalyst for imine reduction*: A mixture of imine (25 mg), catalyst (1 mol %) in FA (formic acid)/TEA (triethylamine) (5:2, 0.1 mL) was stirred at 301–333 K for 18–41 h under an inert atmosphere. For reaction monitoring, an aliquot of the reaction mixture was filtered through a plug of silica and analyzed by GC for percentage conversion. Enantiomeric excess and conversion were determined by GC analysis (chrompaccyclodextrin-β-236 M-19, 50 m×0.25 mm×0.25 μm, *T*=443 K, *P*=15 psi, gas H_2_, imine 38.46 min, *S* isomer 35.12 min, *R* isomer 35.94 min).

*Computational details*: Geometry optimization calculations of **1**, **2**, **3**, and **4** and their enantiomers were performed at the DFT level in the gas phase using Gaussian 03.^83^ The Becke three parameter hybrid functional and Lee–Yang–Parr’s gradient corrected correlation functional (B3LYP)^84^, ^85^ were employed together with the LanL2DZ^86^ effective core potential for the Os atom and the 6-31G** basis set for all other atoms.^87^ The nature of all stationary points was confirmed by normal mode analysis.

## References

[b1] De Camp WH (1993). J. Pharm. Biomed. Anal.

[b2] D’Amato RJ, Loughnan MS, Flynn E, Folkman J (1994). Proc. Natl. Acad. Sci. USA.

[b3] Singhal S, Mehta J, Desikan R, Ayers D, Roberson P, Eddlemon P, Munshi N, Anaissie E, Wilson C, Dhodapkar M, Zeldis J, Siegel D, Crowley J, Barlogie B (1999). New Engl. J. Med.

[b4] Gasser G, Ott I, Metzler-Nolte N (2011). J. Med. Chem.

[b5] Hartinger CG, Dyson PJ (2009). Chem. Soc. Rev.

[b6] Barry NPE, Sadler PJ (2012). Chem. Soc. Rev.

[b7] Hindo SS, Mancino AM, Braymer JJ, Liu Y, Vivekanandan S, Ramamoorthy A, Lim MH (2009). J. Am. Chem. Soc.

[b8] Ronconi L, Sadler PJ (2007). Coord. Chem. Rev.

[b9] Moreno V, Font-Bardia M, Calvet T, Lorenzo J, Avilés FX, Garcia MH, Morais TS, Valente A, Robalo MP (2011). J. Inorg. Biochem.

[b10] Hanif M, Meier SM, Kandioller W, Bytzek A, Hejl M, Hartinger CG, Nazarov AA, Arion VB, Jakupec MA, Dyson PJ, Keppler BK (2011). J. Inorg. Biochem.

[b11] Caruso F, Rossi M, Benson A, Opazo C, Freedman D, Monti E, Gariboldi MB, Shaulky J, Marchetti F, Pettinari R, Pettinari C (2012). J. Med. Chem.

[b12] Noffke AL, Habtemariam A, Pizarro AM, Sadler PJ (2012). Chem. Commun.

[b13] Fu Y, Habtemariam A, Basri AMBH, Braddick D, Clarkson GJ, Sadler PJ (2011). Dalton Trans.

[b14] Fu Y, Romero MJ, Habtemariam A, Snowden ME, Song L, Clarkson GJ, Qamar B, Pizarro AM, Unwin PR, Sadler PJ (2012). Chem. Sci.

[b15] Rijt SHv, Kostrhunova H, Brabec V, Sadler PJ (2011). Bioconjugate Chem.

[b16] Kurzwernhart A, Kandioller W, Bartel C, Bachler S, Trondl R, Muhlgassner G, Jakupec MA, Arion VB, Marko D, Keppler BK, Hartinger CG (2012). Chem. Commun.

[b17] Stepanenko IN, Casini A, Edafe F, Novak MS, Arion VB, Dyson PJ, Jakupec MA, Keppler BK (2011). Inorg. Chem.

[b18] Ginzinger W, Mühlgassner G, Arion VB, Jakupec MA, Roller A, Galanski M, Reithofer M, Berger W, Keppler BK (2012). J. Med. Chem.

[b19] Sava G, Jaouen G, Hillard EA, Bergamo A (2012). Dalton Trans.

[b20] Paira P, Chow MJ, Venkatesan G, Kosaraju VK, Cheong SL, Klotz K-N, Ang WH, Pastorin G (2013). Chem. Eur. J.

[b21] Atilla-Gokcumen G, Di Costanzo L, Meggers E (2011). J. Biol. Inorg. Chem.

[b22] Blanck S, Geisselbrecht Y, Kraling K, Middel S, Mietke T, Harms K, Essen L-O, Meggers E (2012). Dalton Trans.

[b23] Blanck S, Maksimoska J, Baumeister J, Harms K, Marmorstein R, Meggers E (2012). Angew. Chem.

[b23aa] Blanck S, Maksimoska J, Baumeister J, Harms K, Marmorstein R, Meggers E (2012). Angew. Chem. Int. Ed.

[b24] Brunner H (1969). Angew. Chem.

[b24aa] Brunner H (1969). Angew. Chem. Int. Ed. Engl.

[b25] Brunner H, Aclasis J, Langer M, Steger W (1974). Angew. Chem.

[b25aa] Brunner H, Aclasis J, Langer M, Steger W (1974). Angew. Chem. Int. Ed. Engl.

[b26] Brunner H, Oeschey R, Nuber B (1996). Organometallics.

[b27] Mendoza-Ferri M-G, Hartinger CG, Eichinger RE, Stolyarova N, Severin K, Jakupec MA, Nazarov AA, Keppler BK (2008). Organometallics.

[b28] Carmona D, Viguri F, Pilar Lamata M, Ferrer J, Bardají, Lahoz FJ, García-Orduña P, Oro LA (2012). Dalton Trans.

[b29] Brunner H, Zwack T, Zabel M, Beck W, Böhm A (2003). Organometallics.

[b30] Smalley KSM, Contractor R, Haass NK, Kulp AN, Atilla-Gokcumen GE, Williams DS, Bregman H, Flaherty KT, Soengas MS, Meggers E, Herlyn M (2007). Cancer Res.

[b31] Kilpin KJ, Cammack SM, Clavel CM, Dyson PJ (2013). Dalton Trans.

[b32] Meier SM, Hanif M, Adhireksan Z, Pichler V, Novak M, Jirkovsky E, Jakupec MA, Arion VB, Davey CA, Keppler BK, Hartinger CG (2013). Chem. Sci.

[b33] Boff B, Gaiddon C, Pfeffer M (2013). Inorg. Chem.

[b34] Filak LK, Göschl S, Heffeter P, Ghannadzadeh Samper K, Egger AE, Jakupec MA, Keppler BK, Berger W, Arion VB (2013). Organometallics.

[b35] Stepanenko IN, Krokhin AA, John RO, Roller A, Arion VB, Jakupec MA, Keppler BK (2008). Inorg. Chem.

[b36] Büchel GE, Stepanenko IN, Hejl M, Jakupec MA, Keppler BK, Heffeter P, Berger W, Arion VB (2012). J. Inorg. Biochem.

[b37] Ni W-X, Man W-L, Yiu S-M, Ho M, Cheung MT-W, Ko C-C, Che C-M, Lam Y-W, Lau T-C (2012). Chem. Sci.

[b38] Naota T, Takaya H, Murahashi S-I (1998). Chem. Rev.

[b39] Noyori R, Hashiguchi S (1997). Acc. Chem. Res.

[b40] Wang C, Villa-Marcos B, Xiao J (2011). Chem. Commun.

[b41] McSkimming A, Colbran SB (2013). Chem. Soc. Rev.

[b42] Faller JW, Lavoie AR (2002). Organometallics.

[b43] Faller JW, Lavoie AR (2001). Org. Lett.

[b44] Wainer IW, Granvil CP (1993). Ther. Drug Monit.

[b45] Nieto S, Dragna JM, Anslyn EV (2010). Chem. Eur. J.

[b46] Peacock AFA, Sadler PJ (2008). Chem. Asian J.

[b47] Lecomte C, Dusausoy Y, Protas J, Tirouflet J, Dormond A (1974). J. Organomet. Chem.

[b48] Stanley K, Baird MC (1975). J. Am. Chem. Soc.

[b49] An H-Y, Wang E-B, Xiao D-R, Li Y-G, Su Z-M, Xu L (2006). Angew. Chem.

[b49aa] An H-Y, Wang E-B, Xiao D-R, Li Y-G, Su Z-M, Xu L (2006). Angew. Chem. Int. Ed.

[b50] Brunner H, Muschiol M, Tsuno T, Takahashi T, Zabel M (2010). Organometallics.

[b51] Brunner H (1999). Angew. Chem.

[b51aa] Brunner H (1999). Angew. Chem. Int. Ed.

[b52] Chen H, Parkinson JA, Nováková O, Bella J, Wang F, Dawson A, Gould R, Parsons S, Brabec V, Sadler PJ (2003). Proc. Natl. Acad. Sci. USA.

[b53] Ganter C (2003). Chem. Soc. Rev.

[b54] Brunner H, Zwack T (2000). Organometallics.

[b55] Consiglio G, Morandini F (1987). Chem. Rev.

[b56] Shnyder SD, Fu Y, Habtemariam A, van Rijt SH, Cooper PA, Loadman PM, Sadler PJ (2011). MedChemComm.

[b57] Fu Y, Habtemariam A, Pizarro AM, van Rijt SH, Healey DJ, Cooper PA, Shnyder SD, Clarkson GJ, Sadler PJ (2010). J. Med. Chem.

[b58] Hotze ACG, Kariuki BM, Hannon MJ (2006). Angew. Chem..

[b58aa] Hotze ACG, Kariuki BM, Hannon MJ (2006). Angew. Chem. Int. Ed.

[b59] McDonnell U, Kerchoffs JMCA, Castiñeiras RPM, Hicks MR, Hotze ACG, Hannon MJ, Rodger A (2008). Dalton Trans.

[b60] Velders AH, van der Schilden K, Hotze ACG, Reedijk J, Kooijman H, Spek AL (2004). Dalton Trans.

[b61] Hotze A, Caspers S, Vos D, Kooijman H, Spek A, Flamigni A, Bacac M, Sava G, Haasnoot J, Reedijk J (2004). J. Biol. Inorg. Chem.

[b62] Dougan SJ, Habtemariam A, McHale SE, Parsons S, Sadler PJ (2008). Proc. Natl. Acad. Sci. USA.

[b63] Paull KD, Shoemaker RH, Hodes L, Monks A, Scudiero DA, Rubinstein L, Plowman J, Boyd MR (1989). J. Natl. Cancer Inst.

[b64] Holbeck SL, Collins JM, Doroshow JH (2010). Mol. Cancer Ther.

[b65] Mao J, Baker DC (1999). Org. Lett.

[b66] Wang C, Pettman A, Bacsa J, Xiao J (2010). Angew. Chem.

[b66aa] Wang C, Pettman A, Bacsa J, Xiao J (2010). Angew. Chem. Int. Ed.

[b67] Carmona D, Lahoz FJ, García-Orduña P, Oro LA, Lamata MP, Viguri F (2012). Organometallics.

[b68] Carmona D, Vega C, Lahoz FJ, Elipe S, Oro LA, Lamata MP, Viguri F, García-Correas R, Cativiela C, López-Ram de Víu MP (1999). Organometallics.

[b69] Soni R, Cheung FK, Clarkson GC, Martins JED, Graham MA, Wills M (2011). Org. Biomol. Chem.

[b70] Ringenberg M, Ward TR (2011). Chem. Commun.

[b71] Rath RK, Nethaji M, Chakravarty AR (2002). Polyhedron.

[b72] Rath RK, Gowda GAN, Chakravarty AR (2002). Proc. Indian Acad. Sci.

[b73] Brunner H, Zwack T, Zabel M (2003). Polyhedron.

[b74] Liu H-K, Sadler PJ (2011). Acc. Chem. Res.

[b75] Chan J, Khan SN, Harvey I, Merrick W, Pelletier J (2004). RNA.

[b76] Shoemaker RH (2006). Nat. Rev. Cancer.

[b77] Lee JK, Havaleshko DM, Cho H, Weinstein JN, Kaldjian EP, Karpovich J, Grimshaw A, Theodorescu D (2007). Proc. Natl. Acad. Sci. USA.

[b78] Yeh HJC, Chrzanowska M, Brossi A (1988). FEBS Lett.

[b79] Görmen M, Pigeon P, Top S, Hillard EA, Huché M, Hartinger CG, de Montigny F, Plamont M-A, Vessières A, Jaouen G (2010). ChemMedChem.

[b80] (2007). CrysAlis PRO.

[b81] Sheldrick GM (1990). Acta Crystallogr. Sect. A.

[b82] Sheldrick GM (1997). SHELX97.

[b83] Frisch MJ, Trucks GW, Schlegel HB, Scuseria GE, Robb MA, Cheeseman JR, Montgomery JA, Vreven T, Kudin KN, Burant JC, Millam JM, Iyengar SS, Tomasi J, Barone V, Mennucci B, Cossi M, Scalmani G, Rega N, Petersson GA, Nakatsuji H, Hada M, Ehara M, Toyota K, Fukuda R, Hasegawa J, Ishida M, Nakajima T, Honda Y, Kitao O, Nakai H, Klene M, Li X, Knox JE, Hratchian HP, Cross JB, Bakken V, Adamo C, Jaramillo J, Gomperts R, Stratmann RE, Yazyev O, Austin AJ, Cammi R, Pomelli C, Ochterski JW, Ayala PY, Morokuma K, Voth GA, Salvador P, Dannenberg JJ, Zakrzewski VG, Dapprich S, Daniels AD, Strain MC, Farkas O, Malick DK, Rabuck AD, Raghavachari K, Foresman JB, Ortiz JV, Cui Q, Baboul AG, Clifford S, Cioslowski J, Stefanov BB, Liu G, Liashenko A, Piskorz P, Komaromi I, Martin RL, Fox DJ, Keith T, Al-Laham MA, Peng CY, Nanayakkara A, Challacombe M, Gill PMW, Johnson B, Chen W, Wong MW, Gonzalez C, Pople JA (2004). Gaussian, Inc..

[b84] Becke AD (1993). J. Chem. Phys.

[b85] Lee C, Yang W, Parr RG (1988). Phys. Rev. B.

[b86] Hay PJ, Wadt WR (1985). J. Chem. Phys.

[b87] McLean AD, Chandler GS (1980). J. Chem. Phys.

