# Supplementary material for: Mirror-Image Organometallic Osmium Arene Iminopyridine Halido Complexes Exhibit Similar Potent Anticancer Activity
Source: Chemistry. 2013 Sep 23;19(45):15199–209. doi: 10.1002/chem.201302183 (PMC4280897; doi:10.1002/chem.201302183)
Supplement: Supplementary file 1 [file chem0019-15199-sd1.pdf]

CHEMISTRY
A EUROPEAN JOURNAL

Supporting Information

© Copyright Wiley-VCH Verlag GmbH & Co. KGaA, 69451 Weinheim, 2013

Mirror-Image Organometallic Osmium Arene Iminopyridine Halido Complexes Exhibit Similar Potent Anticancer Activity

Ying Fu,[a] Rina Soni,[a] María J. Romero,[a] Ana M. Pizarro,[a] Luca Salassa,[a, b] Guy J. Clarkson,[a] Jessica M. Hearn,[a, c] Abraha Habtemariam,[a] Martin Wills,[a] and Peter J. Sadler*[a]

chem_201302183_sm_miscellaneous_information.pdf

SUPPORTING INFORMATION

Contents:

Tables S1-S4
Figures S1-S5

Table S1. Anticancer activity in the NCI 60-cell line screen. (A) complex **2** (NSC: D-758116/1), (B) complex **4** (NSC: D-758118/1). GI_{50} = IC_{50}: the concentration that inhibits cell growth by 50%.

(A) Complex 2

National Cancer Institute Developmental Therapeutics Program
In-Vitro Testing Results

NSC : D - 758116 / 1	Experiment ID : 1106NS66	Test Type : 08	Units : Molar
Report Date : August 29, 2011	Test Date : June 20, 2011	QNS :	MC :
COMI : FY175A (105701)	Stain Reagent : SRB Dual-Pass Related	SSPL : 0Y4T	

			Log10 Concentration												
	Time		Mean Optical Densities					Percent Growth							
Panel/Cell Line	Zero	Ctrl	-8.0	-7.0	-6.0	-5.0	-4.0	-8.0	-7.0	-6.0	-5.0	-4.0	GI50	TGI	LC50
Leukemia															
HL-60(TB)	0.836	2.721	2.765	2.886	2.834	1.095	0.503	102	109	106	14	-40	4.04E-6	1.80E-5	> 1.00E-4
MOLT-4	0.495	2.013	2.008	2.002	2.010	1.558	0.589	100	99	100	70	6	2.06E-5	> 1.00E-4	> 1.00E-4
SR	0.409	1.836	1.688	1.633	1.554	1.025	0.422	90	86	80	43	1	6.54E-6	> 1.00E-4	> 1.00E-4
Non-Small Cell Lung Cancer															
A549/ATCC	0.264	1.402	1.384	1.301	1.247	0.867	0.491	98	91	86	53	20	1.23E-5	> 1.00E-4	> 1.00E-4
EKVX	0.774	1.837	1.803	1.728	1.658	1.202	0.936	97	90	83	40	15	5.91E-6	> 1.00E-4	> 1.00E-4
HOP-62	0.479	1.362	1.362	1.364	1.308	0.827	0.618	100	100	94	39	16	6.38E-6	> 1.00E-4	> 1.00E-4
HOP-92	1.062	1.466	1.470	1.494	1.391	1.086	0.617	101	107	82	6	-42	2.61E-6	1.32E-5	> 1.00E-4
NCI-H226	0.558	1.441	1.391	1.377	1.363	1.172	0.806	94	93	91	70	28	2.95E-5	> 1.00E-4	> 1.00E-4
NCI-H23	0.486	1.697	1.690	1.677	1.661	1.074	0.575	99	98	97	49	7	9.33E-6	> 1.00E-4	> 1.00E-4
NCI-H322M	0.705	1.703	1.594	1.583	1.634	1.608	0.979	89	88	93	90	27	4.38E-5	> 1.00E-4	> 1.00E-4
NCI-H460	0.246	2.527	2.635	2.638	2.522	0.823	0.361	105	105	100	25	5	4.66E-6	> 1.00E-4	> 1.00E-4
NCI-H522	0.526	1.267	1.219	1.262	1.176	0.603	0.240	94	99	88	10	-54	3.07E-6	1.45E-5	8.56E-5
Colon Cancer															
COLO 205	0.240	1.400	1.447	1.396	1.096	0.080	0.084	104	100	74	-67	-65	1.48E-6	3.35E-6	7.59E-6
HCC-2998	0.539	2.052	1.939	1.948	1.874	1.053	0.573	93	93	88	34	2	5.06E-6	> 1.00E-4	> 1.00E-4
HCT-116	0.226	1.675	1.716	1.714	1.576	0.577	0.303	103	103	93	24	5	4.23E-6	> 1.00E-4	> 1.00E-4
HCT-15	0.301	1.793	1.843	1.864	1.733	1.843	1.545	103	105	96	103	83	> 1.00E-4	> 1.00E-4	> 1.00E-4
HT29	0.175	1.115	1.159	1.140	1.133	0.529	0.200	105	103	102	38	3	6.42E-6	> 1.00E-4	> 1.00E-4
KM12	0.441	2.391	2.421	2.443	2.344	1.007	0.570	102	103	98	29	7	4.95E-6	> 1.00E-4	> 1.00E-4
SW-620	0.199	1.349	1.370	1.373	1.317	0.747	0.329	102	102	97	48	11	8.96E-6	> 1.00E-4	> 1.00E-4
CNS Cancer															
SF-268	0.360	1.296	1.300	1.311	1.332	0.752	0.521	100	102	104	42	17	7.38E-6	> 1.00E-4	> 1.00E-4
SF-295	0.860	2.494	2.359	2.291	2.350	2.272	1.010	92	88	91	86	9	2.96E-5	> 1.00E-4	> 1.00E-4
SF-539	0.572	1.550	1.613	1.598	1.591	1.049	0.696	106	105	104	49	13	9.49E-6	> 1.00E-4	> 1.00E-4
SNB-19	0.503	1.641	1.542	1.529	1.466	0.858	0.736	91	90	85	31	20	4.45E-6	> 1.00E-4	> 1.00E-4
SNB-75	0.771	1.288	1.213	1.195	1.220	0.813	0.527	85	82	87	8	-32	2.94E-6	1.60E-5	> 1.00E-4
U251	0.239	1.064	1.056	1.043	0.960	0.481	0.325	99	97	87	29	10	4.40E-6	> 1.00E-4	> 1.00E-4
Melanoma															
LOX IMVI	0.214	1.406	1.377	1.309	1.269	0.536	0.228	98	92	89	27	1	4.23E-6	> 1.00E-4	> 1.00E-4
MALME-3M	0.626	1.007	0.974	0.962	0.928	0.734	0.414	91	88	79	28	-34	3.74E-6	2.85E-5	> 1.00E-4
M14	0.405	1.617	1.647	1.692	1.609	1.310	0.457	102	106	99	75	4	2.24E-5	> 1.00E-4	> 1.00E-4
MDA-MB-435	0.416	2.005	1.945	1.872	1.778	0.637	0.266	96	92	86	14	-36	3.14E-6	1.90E-5	> 1.00E-4
SK-MEL-2	0.883	1.929	1.986	2.067	1.976	1.096	0.344	105	113	105	20	-61	4.44E-6	1.78E-5	7.31E-5
SK-MEL-28	0.484	1.298	1.294	1.318	1.238	0.865	0.311	100	102	93	47	-36	8.50E-6	3.69E-5	> 1.00E-4
SK-MEL-5	0.517	2.279	2.208	2.134	1.938	0.394	0.009	96	92	81	-24	-98	1.96E-6	5.91E-6	2.24E-5
UACC-257	0.628	1.415	1.362	1.384	1.256	0.683	0.396	93	96	80	7	-37	2.57E-6	1.44E-5	> 1.00E-4
UACC-62	0.701	2.474	2.328	2.298	2.130	1.201	0.221	92	90	81	28	-69	3.83E-6	1.96E-5	6.43E-5
Ovarian Cancer															
IGROV1	0.425	1.468	1.502	1.445	1.402	0.933	0.393	103	98	94	49	-8	9.34E-6	7.35E-5	> 1.00E-4
OVCAR-3	0.455	1.472	1.518	1.492	1.338	0.676	0.489	105	102	87	22	3	3.68E-6	> 1.00E-4	> 1.00E-4
OVCAR-4	0.677	1.724	1.711	1.760	1.571	0.827	0.672	99	103	85	14	-1	3.14E-6	8.93E-5	> 1.00E-4
OVCAR-5	0.487	1.148	1.157	1.145	1.106	0.984	0.561	101	100	94	75	11	2.48E-5	> 1.00E-4	> 1.00E-4
OVCAR-8	0.321	1.343	1.309	1.331	1.304	0.693	0.408	97	99	96	36	8	5.92E-6	> 1.00E-4	> 1.00E-4
NCI/ADR-RES	0.467	1.528	1.507	1.501	1.504	1.534	1.548	98	97	98	101	102	> 1.00E-4	> 1.00E-4	> 1.00E-4
SK-OV-3	0.485	1.258	1.201	1.222	1.233	1.156	0.695	93	95	97	87	27	4.14E-5	> 1.00E-4	> 1.00E-4
Renal Cancer															
786-0	0.595	1.950	1.957	1.973	1.896	2.023	0.952	100	102	96	105	26	5.02E-5	> 1.00E-4	> 1.00E-4
A498	1.041	2.028	2.013	2.027	1.995	1.970	1.516	98	100	97	94	48	9.12E-5	> 1.00E-4	> 1.00E-4
ACHN	0.410	1.630	1.587	1.681	1.570	1.543	1.234	96	104	95	93	68	> 1.00E-4	> 1.00E-4	> 1.00E-4
CAKI-1	0.760	2.257	2.189	2.162	2.167	2.231	2.275	95	94	94	98	101	> 1.00E-4	> 1.00E-4	> 1.00E-4
RXF 393	0.282	0.749	0.744	0.751	0.722	0.698	0.329	99	101	94	89	10	3.12E-5	> 1.00E-4	> 1.00E-4
SN12C	0.524	1.993	1.834	1.898	1.771	1.089	0.676	89	94	85	38	10	5.64E-6	> 1.00E-4	> 1.00E-4
TK-10	0.583	1.405	1.440	1.443	1.466	1.368	0.861	104	105	107	95	34	5.46E-5	> 1.00E-4	> 1.00E-4
UO-31	0.535	1.488	1.374	1.324	1.333	1.422	1.399	88	83	84	93	91	> 1.00E-4	> 1.00E-4	> 1.00E-4
Prostate Cancer															
PC-3	0.493	1.665	1.664	1.620	1.542	0.797	0.550	100	96	90	26	5	4.18E-6	> 1.00E-4	> 1.00E-4
DU-145	0.410	1.631	1.710	1.667	1.710	1.146	0.766	106	103	106	60	29	2.14E-5	> 1.00E-4	> 1.00E-4
Breast Cancer															
MCF7	0.549	2.170	2.092	2.092	2.102	0.918	0.640	95	95	96	23	6	4.24E-6	> 1.00E-4	> 1.00E-4
MDA-MB-231/ATCC	0.465	1.121	1.095	1.121	1.089	0.766	0.366	96	100	95	46	-21	8.24E-6	4.82E-5	> 1.00E-4
HS 578T	0.722	1.541	1.559	1.566	1.584	1.368	0.958	102	103	105	79	29	3.77E-5	> 1.00E-4	> 1.00E-4
BT-549	0.750	1.565	1.583	1.583	1.566	0.986	0.709	102	102	100	29	-5	5.06E-6	6.94E-5	> 1.00E-4
T-47D	0.518	1.169	1.173	1.167	1.069	0.692	0.580	101	100	85	27	10	3.96E-6	> 1.00E-4	> 1.00E-4
MDA-MB-468	0.569	1.477	1.397	1.354	0.963	0.508	0.163	91	87	43	-11	-71	7.03E-7	6.32E-6	4.44E-5

(B) Complex 4

National Cancer Institute Developmental Therapeutics Program<br>In-Vitro Testing Results			
NSC : D - 758118 / 1	Experiment ID : 1106NS66	Test Type : 08	Units : Molar
Report Date : August 29, 2011	Test Date : June 20, 2011	QNS :	MC :
COMI : FY178A (105704)	Stain Reagent : SRB Dual-Pass Related	SSPL : 0Y4T	

			Log10 Concentration												
	Time		Mean Optical Densities					Percent Growth							
Panel/Cell Line	Zero	Ctrl	-8.0	-7.0	-6.0	-5.0	-4.0	-8.0	-7.0	-6.0	-5.0	-4.0	GI50	TGI	LC50
**Leukemia**															
HL-60(TB)	0.836	2.696	2.687	2.602	2.287	0.912	0.397	99	95	78	4	-53	2.39E-6	1.18E-5	9.01E-5
MOLT-4	0.495	1.972	1.919	1.903	1.838	1.168	0.629	96	95	91	46	9	7.99E-6	> 1.00E-4	> 1.00E-4
SR	0.409	1.757	1.686	1.574	1.499	0.594	0.375	95	86	81	14	-8	2.88E-6	4.19E-5	> 1.00E-4
**Non-Small Cell Lung Cancer**															
A549/ATCC	0.264	1.381	1.317	1.302	1.316	0.737	0.414	94	93	94	42	13	7.12E-6	> 1.00E-4	> 1.00E-4
EKVX	0.774	1.827	1.806	1.709	1.589	1.113	0.945	98	89	77	32	16	4.03E-6	> 1.00E-4	> 1.00E-4
HOP-62	0.479	1.320	1.291	1.270	1.281	0.788	0.494	97	94	95	37	2	5.94E-6	> 1.00E-4	> 1.00E-4
HOP-92	1.062	1.449	1.462	1.429	1.433	0.911	0.547	103	95	96	-14	-49	2.61E-6	7.43E-6	> 1.00E-4
NCI-H226	0.558	1.452	1.397	1.393	1.363	1.144	0.777	94	93	90	66	25	2.39E-5	> 1.00E-4	> 1.00E-4
NCI-H23	0.486	1.662	1.616	1.612	1.580	0.858	0.524	96	96	93	32	3	5.02E-6	> 1.00E-4	> 1.00E-4
NCI-H322M	0.705	1.773	1.705	1.635	1.627	1.491	0.870	94	87	86	74	15	2.55E-5	> 1.00E-4	> 1.00E-4
NCI-H460	0.246	2.506	2.649	2.590	2.495	0.812	0.356	106	104	100	25	5	4.62E-6	> 1.00E-4	> 1.00E-4
NCI-H522	0.526	1.261	1.185	1.241	1.057	0.542	0.185	90	97	72	2	-65	2.07E-6	1.08E-5	6.01E-5
**Colon Cancer**															
COLO 205	0.240	1.309	1.309	1.310	1.071	0.065	0.083	100	100	78	-73	-66	1.53E-6	3.28E-6	7.04E-6
HCC-2998	0.539	1.834	1.785	1.829	1.731	0.866	0.471	96	100	92	25	-13	4.26E-6	4.64E-5	> 1.00E-4
HCT-116	0.226	1.735	1.775	1.805	1.696	0.552	0.283	103	105	97	22	4	4.22E-6	> 1.00E-4	> 1.00E-4
HCT-15	0.301	1.877	1.769	1.862	1.874	1.881	1.689	93	99	100	100	88	> 1.00E-4	> 1.00E-4	> 1.00E-4
HT29	0.175	1.073	1.081	1.132	1.076	0.376	0.178	101	107	100	22	.	4.42E-6	> 1.00E-4	> 1.00E-4
KM12	0.441	2.358	2.398	2.440	2.208	0.907	0.554	102	104	92	24	6	4.18E-6	> 1.00E-4	> 1.00E-4
SW-620	0.199	1.345	1.363	1.338	1.327	0.692	0.377	102	99	98	43	16	7.47E-6	> 1.00E-4	> 1.00E-4
**CNS Cancer**															
SF-268	0.360	1.254	1.301	1.330	1.282	0.636	0.449	105	109	103	31	10	5.43E-6	> 1.00E-4	> 1.00E-4
SF-295	0.860	2.358	2.257	2.195	2.225	2.128	0.956	93	89	91	85	6	2.77E-5	> 1.00E-4	> 1.00E-4
SF-539	0.572	1.618	1.663	1.643	1.636	0.933	0.691	104	102	102	34	11	5.88E-6	> 1.00E-4	> 1.00E-4
SNB-19	0.503	1.708	1.646	1.643	1.518	0.843	0.737	95	95	84	28	19	4.09E-6	> 1.00E-4	> 1.00E-4
SNB-75	0.771	1.244	1.165	1.215	1.149	0.736	0.549	83	94	80	-5	-29	2.25E-6	8.83E-6	> 1.00E-4
U251	0.239	1.032	0.982	0.974	0.876	0.400	0.274	94	93	80	20	4	3.20E-6	> 1.00E-4	> 1.00E-4
**Melanoma**															
LOX IMVI	0.214	1.364	1.281	1.305	1.242	0.425	0.205	93	95	89	18	-4	3.58E-6	6.38E-5	> 1.00E-4
MALME-3M	0.626	1.035	1.009	0.974	0.911	0.652	0.350	94	85	70	6	-44	2.04E-6	1.33E-5	> 1.00E-4
M14	0.405	1.590	1.617	1.598	1.542	1.272	0.642	102	101	96	73	20	2.72E-5	> 1.00E-4	> 1.00E-4
MDA-MB-435	0.416	2.000	1.932	1.883	1.678	0.573	0.296	96	93	80	10	-29	2.66E-6	1.80E-5	> 1.00E-4
SK-MEL-2	0.883	1.889	1.949	2.025	1.995	1.013	0.290	106	114	111	13	-67	4.17E-6	1.45E-5	6.11E-5
SK-MEL-28	0.484	1.208	1.223	1.258	1.152	0.782	0.277	102	107	92	41	-43	6.69E-6	3.09E-5	> 1.00E-4
SK-MEL-5	0.517	2.156	2.098	2.089	1.700	0.276	0.006	96	96	72	-47	-99	1.54E-6	4.05E-6	1.16E-5
UACC-257	0.628	1.368	1.282	1.277	1.169	0.607	0.415	88	88	73	-3	-34	2.01E-6	9.02E-6	> 1.00E-4
UACC-62	0.701	2.381	2.278	2.300	2.044	1.134	0.322	94	95	80	26	-54	3.57E-6	2.10E-5	8.89E-5
**Ovarian Cancer**															
IGROV1	0.425	1.457	1.523	1.462	1.364	0.821	0.316	106	100	91	38	-26	6.00E-6	3.97E-5	> 1.00E-4
OVCAR-3	0.455	1.456	1.502	1.527	1.327	0.620	0.442	105	107	87	16	-3	3.35E-6	7.04E-5	> 1.00E-4
OVCAR-4	0.677	1.661	1.795	1.744	1.503	0.794	0.634	114	108	84	12	-6	2.96E-6	4.46E-5	> 1.00E-4
OVCAR-5	0.487	1.201	1.183	1.190	1.188	0.927	0.523	98	99	98	62	5	1.60E-5	> 1.00E-4	> 1.00E-4
OVCAR-8	0.321	1.363	1.300	1.316	1.272	0.779	0.379	94	96	91	44	6	7.44E-6	> 1.00E-4	> 1.00E-4
NCI/ADR-RES	0.467	1.460	1.529	1.468	1.468	1.514	1.497	107	101	101	105	104	> 1.00E-4	> 1.00E-4	> 1.00E-4
SK-OV-3	0.485	1.215	1.191	1.209	1.187	1.033	0.555	97	99	96	75	10	2.41E-5	> 1.00E-4	> 1.00E-4
**Renal Cancer**															
786-0	0.595	1.965	2.003	1.970	1.979	2.056	1.259	103	100	101	107	48	9.39E-5	> 1.00E-4	> 1.00E-4
A498	1.041	2.009	1.921	1.989	2.006	1.956	1.537	91	98	100	94	51	> 1.00E-4	> 1.00E-4	> 1.00E-4
ACHN	0.410	1.618	1.638	1.681	1.662	1.687	1.413	102	105	104	106	83	> 1.00E-4	> 1.00E-4	> 1.00E-4
CAKI-1	0.760	2.236	2.157	2.140	2.130	2.284	2.380	95	93	93	103	110	> 1.00E-4	> 1.00E-4	> 1.00E-4
RXF 393	0.282	0.701	0.704	0.693	0.688	0.685	0.390	101	98	97	96	26	4.53E-5	> 1.00E-4	> 1.00E-4
SN12C	0.524	1.943	1.867	1.888	1.772	0.870	0.645	95	96	88	24	9	3.95E-6	> 1.00E-4	> 1.00E-4
TK-10	0.583	1.358	1.392	1.392	1.390	1.359	0.924	104	104	104	100	44	7.81E-5	> 1.00E-4	> 1.00E-4
UO-31	0.535	1.535	1.389	1.359	1.369	1.421	1.380	85	82	83	89	85	> 1.00E-4	> 1.00E-4	> 1.00E-4
**Prostate Cancer**															
PC-3	0.493	1.626	1.661	1.610	1.367	0.691	0.511	103	99	77	17	2	2.85E-6	> 1.00E-4	> 1.00E-4
DU-145	0.410	1.609	1.735	1.723	1.666	0.999	0.696	111	110	105	49	24	9.65E-6	> 1.00E-4	> 1.00E-4
**Breast Cancer**															
MCF7	0.549	2.063	1.997	2.018	1.945	0.741	0.537	96	97	92	13	-2	3.39E-6	7.13E-5	> 1.00E-4
MDA-MB-231/ATCC	0.465	1.169	1.141	1.129	1.049	0.648	0.333	96	94	83	26	-28	3.78E-6	3.01E-5	> 1.00E-4
HS 578T	0.722	1.577	1.597	1.561	1.592	1.232	0.925	102	98	102	60	24	1.86E-5	> 1.00E-4	> 1.00E-4
BT-549	0.750	1.573	1.614	1.632	1.616	0.918	0.754	105	107	105	20	.	4.48E-6	> 1.00E-4	> 1.00E-4
T-47D	0.518	1.166	1.139	1.108	1.007	0.605	0.514	96	91	75	13	-1	2.57E-6	8.69E-5	> 1.00E-4
MDA-MB-468	0.569	1.483	1.373	1.371	0.895	0.489	0.145	88	88	36	-14	-75	5.30E-7	5.20E-6	3.93E-5

Table S2

COMPARE results using NCI/DTP standard agents database for the IC_{50} endpoint

(S_{Os}, S_c)-[Os(η^6-*p*-cym)(ImpyMe)I]PF_6 (**2**)			(R_{Os}, R_c)-[Os(η^6-*p*-cym)(ImpyMe)I]PF_6 (**4**)		
**Correlated agent**[a]	**PCC**	**Mechanism**	**Correlated agent**[a]	**PCC**	**Mechanism**
Vinblastine sulfate	0.743	Antimicrotubule agent	vinblastine sulfate	0.754	Antimicrotubule agent
phyllanthoside	0.630	Inhibits protein synthesis	macbecin II	0.672	DNA antimetabolite
chromomycin A3	0.624	RNA antimetabolite (binds DNA)	phyllanthoside	0.669	Inhibits protein synthesis
			chromomycin A3	0.640	RNA antimetabolite (binds DNA)
			paclitaxel (Taxol)	0.616	Antimicrotubule agent
			bisantrene hydrochloride	0.613	Topo II inhibitor

[a] NCI/DTP database. Only those compounds with PCC > 0.6 are shown.

Table S3

COMPARE results using NCI/DTP standard agents database for the TGI endpoint

(S_{Os}, S_c)-[Os(η^6-*p*-cym)(ImpyMe)I]PF_6 (**2**)			(R_{Os}, R_c)-[Os(η^6-*p*-cym)(ImpyMe)I]PF_6 (**4**)		
**Correlated agent**[a]	**PCC**	**Mechanism**	**Correlated agent**[a]	**PCC**	**Mechanism**
[b]	[b]	[b]	echinomycin	0.603	DNA intercalator

[a] NCI/DTP database. Only those compounds with PCC > 0.6 are shown.
[b] No correlations with PCC < 0.6

Table S4

COMPARE results using the NCI/DTP standard agents database for the LC_{50} endpoint

(S_{Os}, S_c)-[Os(η^6-*p*-cym)(ImpyMe)I]PF_6 (**2**)			(R_{Os}, R_c)-[Os(η^6-*p*-cym)(ImpyMe)I]PF_6 (**4**)		
**Correlated agent**	**PCC**	**Mechanism**	**Correlated agent**	**PCC**	**Mechanism**
morpholino-ADR	0.946	Topo I inhibitior	morpholino-ADR	0.972	Topo I inhibitior
B-TGDR	0.899	DNA antimetabolite	B-TGDR	0.903	DNA antimetabolite
thioguanine	0.859	DNA antimetabolite	didemnin B	0.898	Inhibits protein synthesis
didemnin B	0.838	Inhibits protein synthesis	tetraplatin	0.857	Alkylating agent
tetraplatin	0.828	Alkylating agent	thioguanine	0.752	DNA antimetabolite
paclitaxel (Taxol)	0.796	Antimicrotubule agent	oxanthrazole	0.748	Topo II inhibitor
bispyridocarbazollum DMS	0.751	NA	paclitaxel (Taxol)	0.738	Antimicrotubule agent
mitramycin	0.740	RNA antimetabolite	vincristine sulfate	0.738	Antimitotic agent
oxanthrazole	0.725	Topo II inhibitor	maytansine	0.695	Antimicrotubule agent
maytansine	0.695	Antimicrotubule agent	mitramycin	0.659	RNA antimetabolite
vincristine sulfate	0.694	Antimitotic agent	chromomycin A3	0.636	RNA antimetabolite (binds DNA)
chromomycin A3	0.678	RNA antimetabolite (binds DNA)	bispyridocarbazollum	0.634	-
topotecan	0.673	Causes double-strand DNA breaks	5-azacytidine	0.608	RNA/DNA antimetabolite
L-asparaginase	0.667	Protein synthesis inhibitor	L-asparaginase	0.606	Protein synthesis inhibitor
vinblastine sulfate	0.659	Antimitotic agent	bactobolin	0.602	Protein synthesis inhibitor
5-azacytidine	0.623	RNA/DNA antimetabolite	vinblastine sulfate	0.601	Antimicrotubule agent
bactobolin	0.612	Protein synthesis inhibitor			

NCI/DTP database. Only those compounds with PCC > 0.6 are shown.

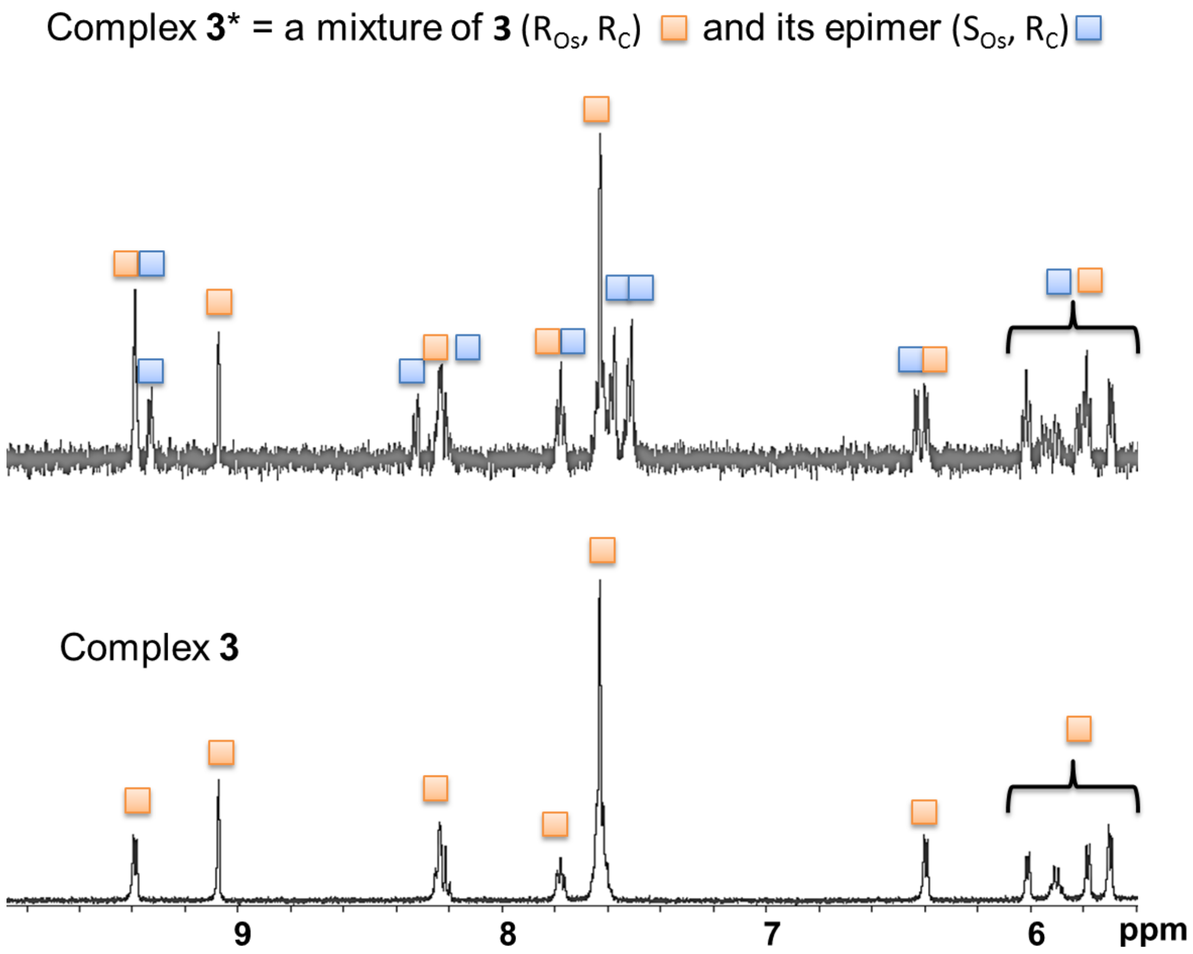

Figure S1. Comparison between the ^{1}H NMR spectra of ((*R*$_{Os}$, *R*$_c$)-[Os(η^6-*p*-cym)(ImpyMe)Cl]PF_6) **3** (red square) and ((*R*$_{Os}$, *R*$_c$) *and* (*S*$_{Os}$, *R*$_c$)-[Os(η^6-*p*-cym)(ImpyMe)Cl]PF_6) **3***(red squares for complex **3** (*R*$_{Os}$, *R*$_c$) and green squares for the epimer of **3** (*S*$_{Os}$, *R*$_c$)) in 10% MeOD/90% D_2O phosphate buffer (pH* 7.4). Orange crystalline **3*** was isolated after concentrating the reaction mixture by evaporating off most of the solvent from the reaction flask.

(A) Complex 1

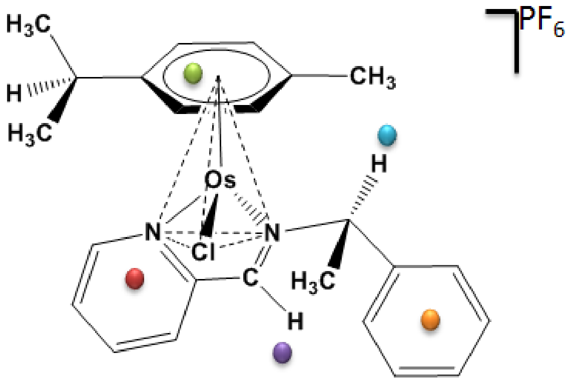

Complex 1: S_{Os}, S_c

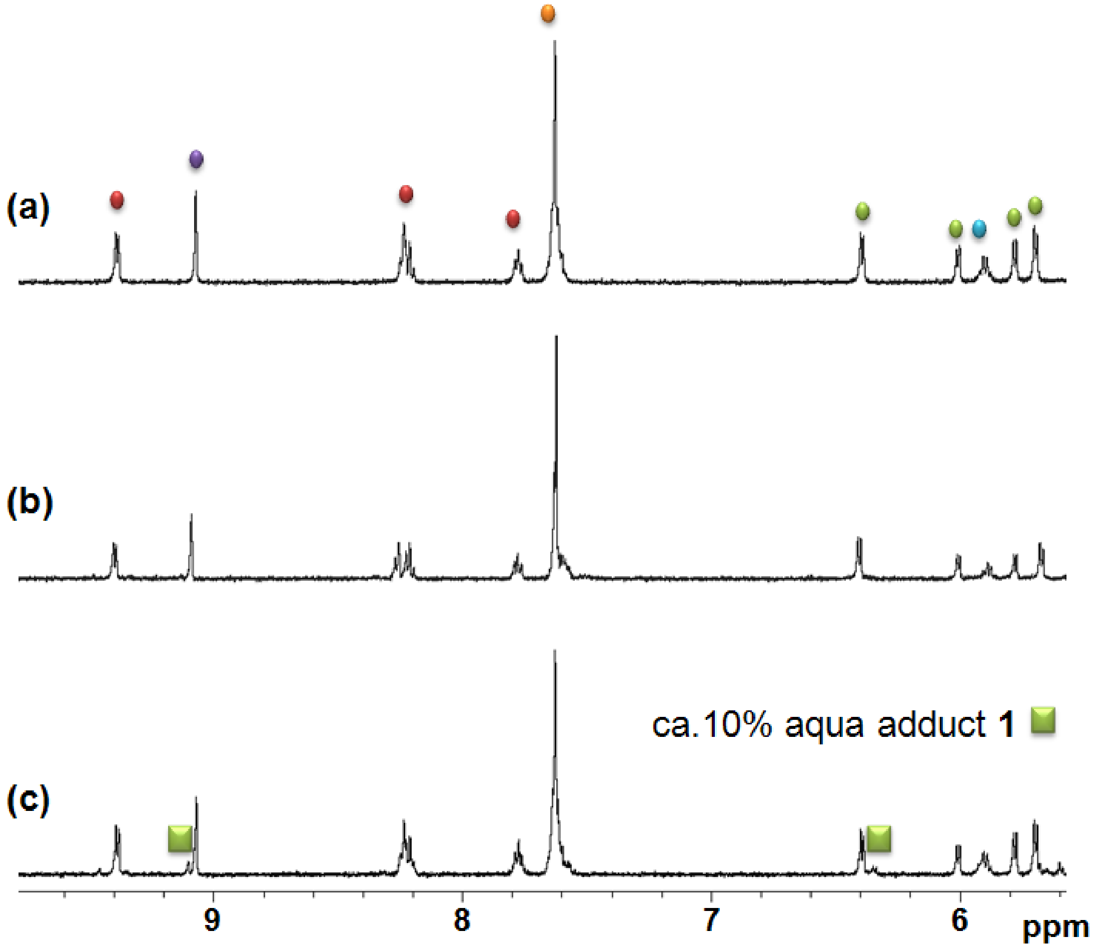

(B) Complex 2

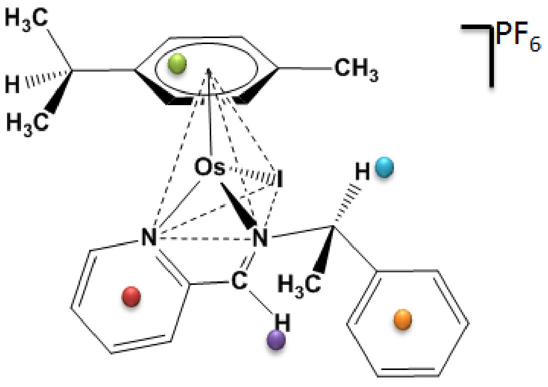

Complex 2: S_{Os}, S_c

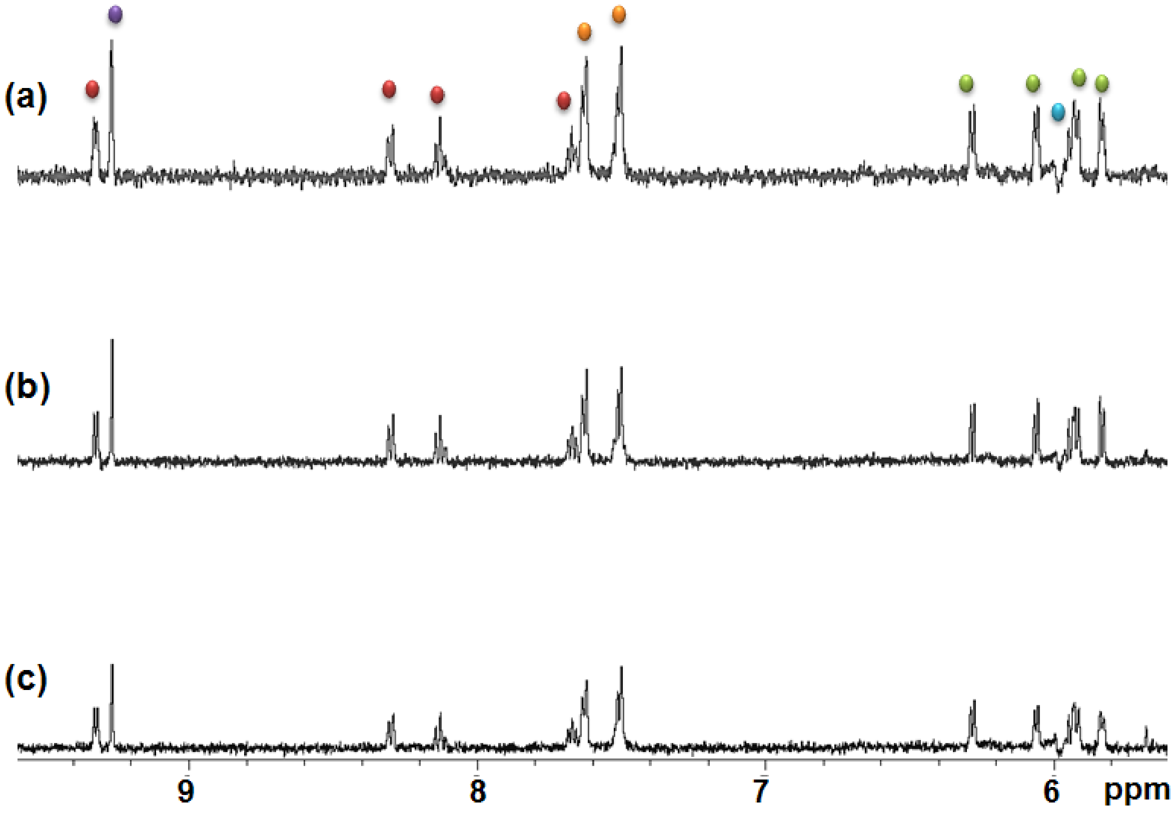

(C) Complex 3

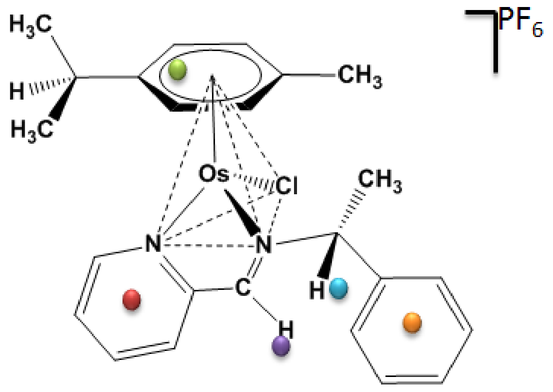

Complex 3: R_{Os}, R_c

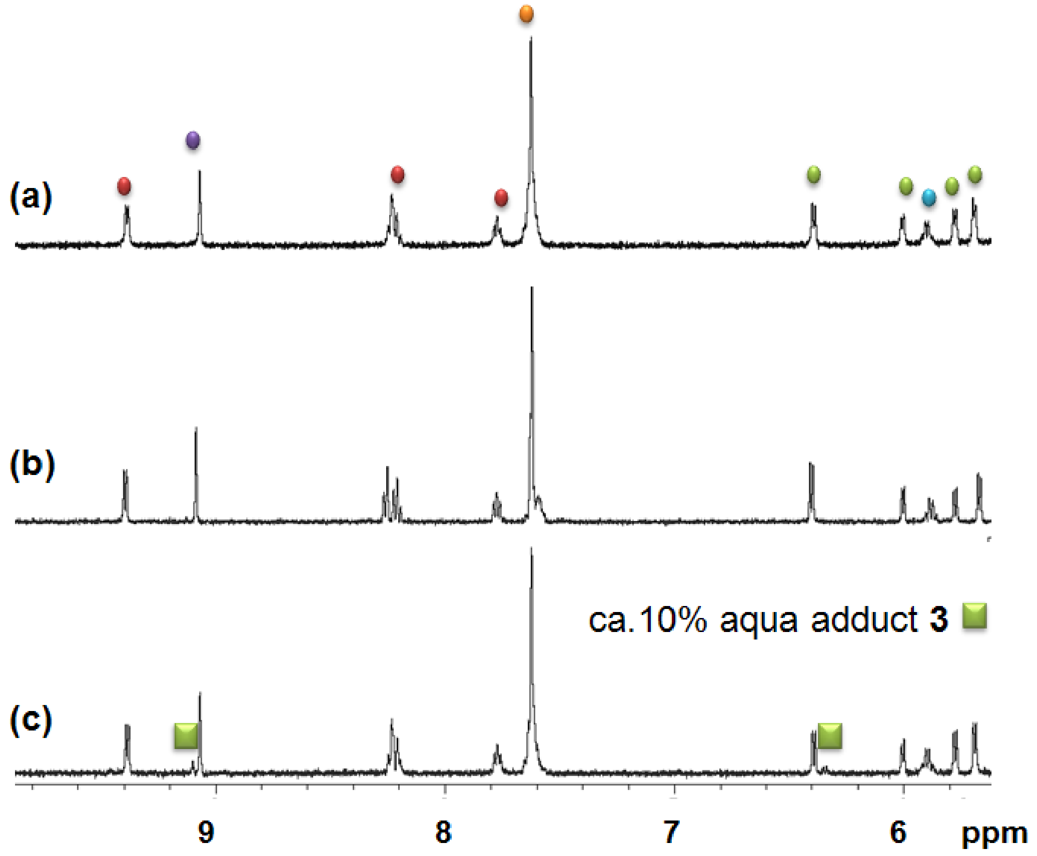

(D) Complex 4

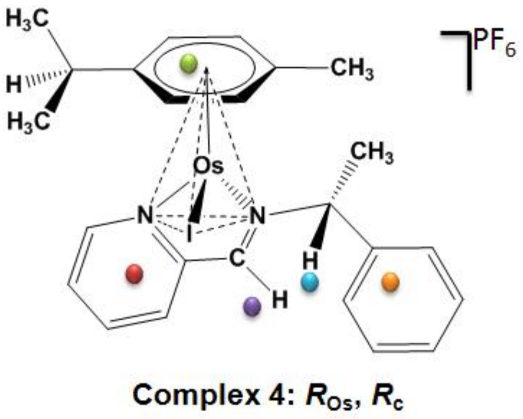

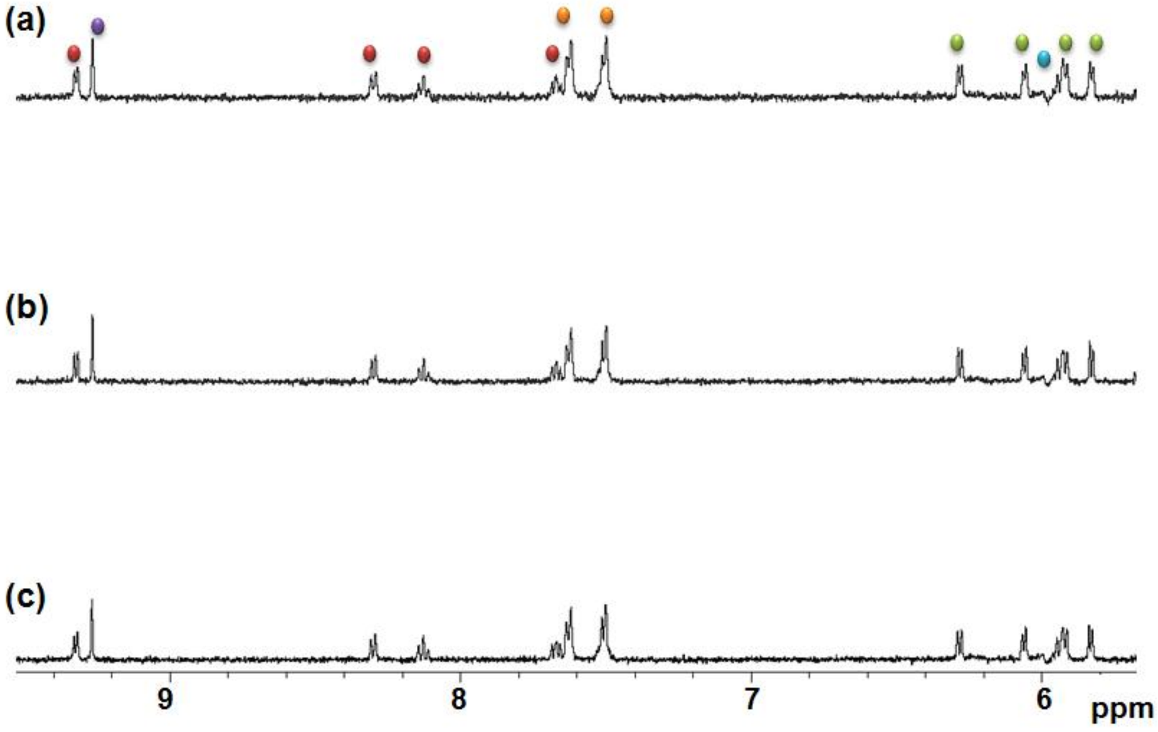

Figure S2. Aromatic region of the ^{1}H NMR spectra of complexes **1** (A), **2** (B), **3** (C), and **4** (D) at a concentration of 100 μM in 10% MeOD/90% D_2O phosphate buffer (pH* 7.4) at t= 0 h and after incubation for 24 h at 310 K under different conditions: **(a)** time 0 h NMR spectra recorded immediately (within 10 min) after preparation of the samples; **(b)** NMR spectra recorded after incubating the samples for 24 h at 310 K with NaCl (500 mM) to suppress aquation, and **(c)** without NaCl.

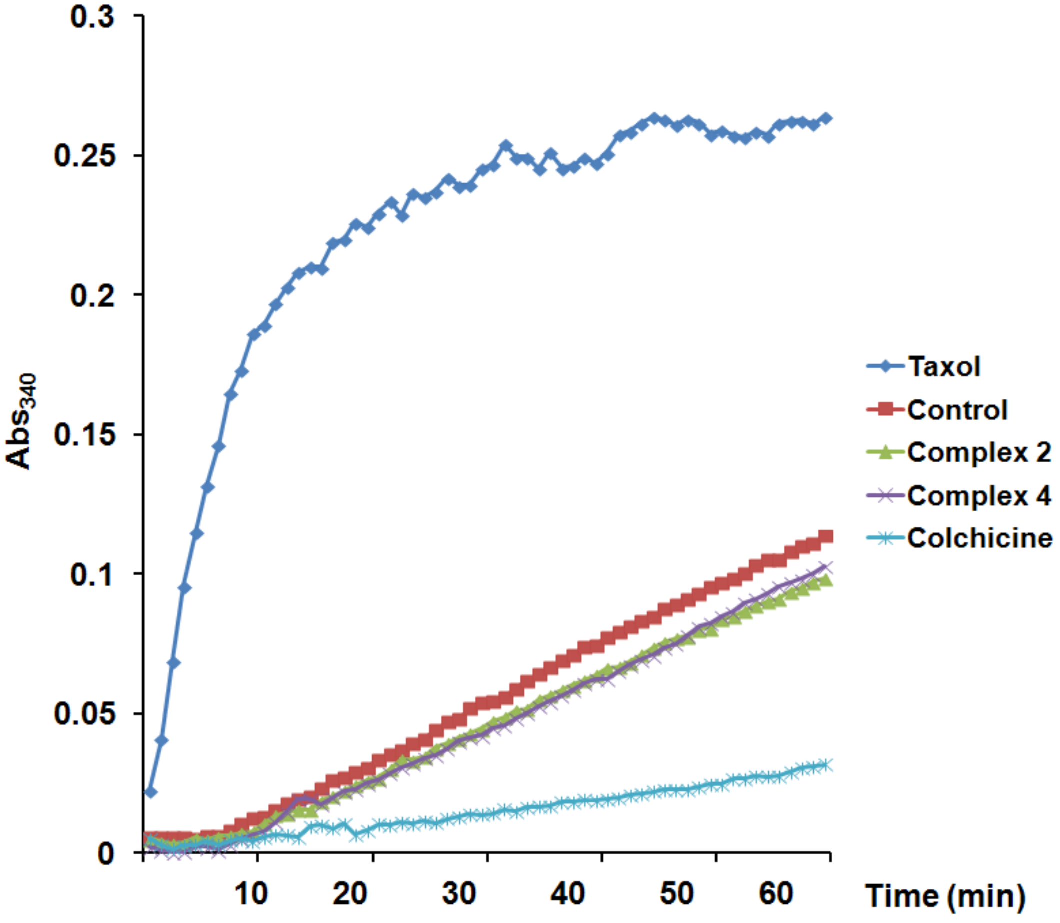

Figure S3. Effects of taxol (facilitator), complex **2**, complex **4** and colchicine (inhibitor) on tubulin polymerization *in vitro*. The absorbance at 340 nm (Abs_{340}) was recorded over a period of 1 hour at 310 K.

(A)

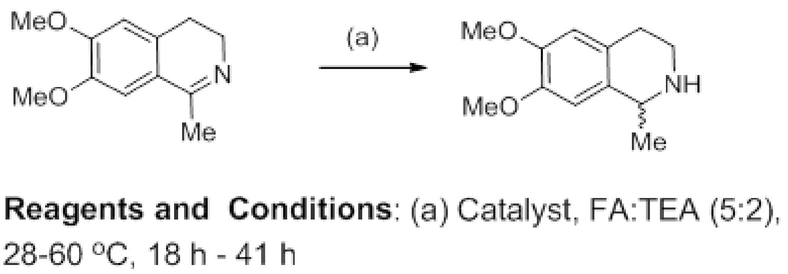

Catalyst	Temperature	Time	% Conv	% ee
**2**	318 K	22 h	40%	22.0% (*R*)
**2**	333 K	18 h	76%	22.0% (*R*)
**4**	301 K	23 h	20%	22.7% (*R*)

(B)

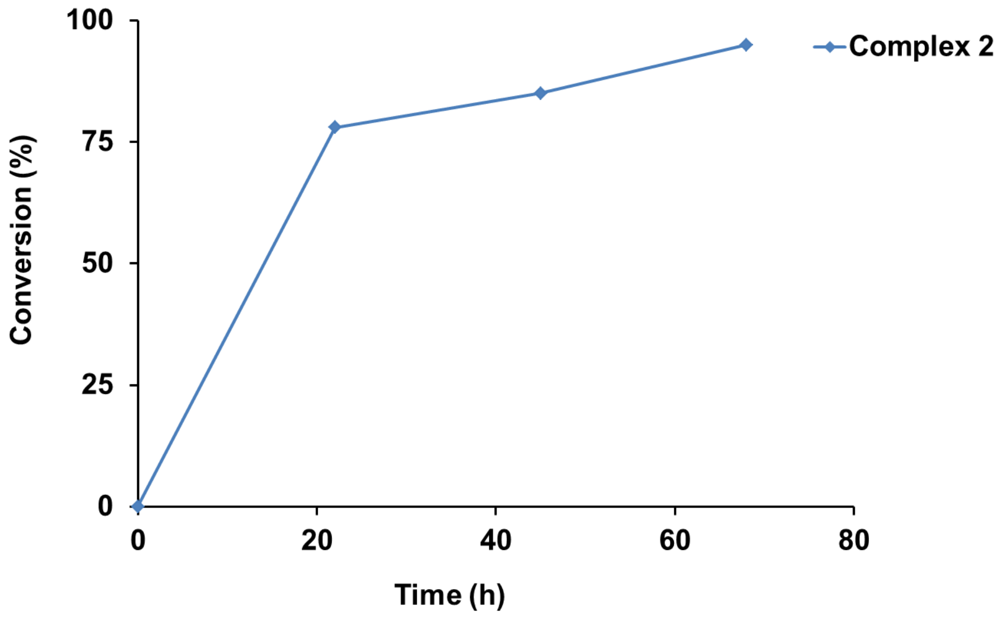

Figure S4. **(A)** Enantiomeric excess and conversions for the imine reduction of (6,7-dimethoxy-1-methyl-3,4-dihydroisoquinoline) by formate catalyzed by **2** and **4** at various temperatures after different periods of time. **(B)** Time dependence of the extent of reduction of the cyclic imine (6,7-dimethoxy-1-methyl-3,4-dihydroisoquinoline) by formate catalyzed by complex **2** at 333 K at different time points. Conditions: imine (25 mg), catalyst (1 mol%) in FA (formic acid) : TEA (triethylamine) = 5:2 (total volume = 0.1 mL).
